# ZRANB2 and SYF2-mediated splicing programs converging on ECT2 are involved in breast cancer cell resistance to doxorubicin

**DOI:** 10.1093/nar/gkz1213

**Published:** 2020-01-16

**Authors:** Iris Tanaka, Alina Chakraborty, Olivier Saulnier, Clara Benoit-Pilven, Sophie Vacher, Dalila Labiod, Eric W F Lam, Ivan Bièche, Olivier Delattre, Frédéric Pouzoulet, Didier Auboeuf, Stéphan Vagner, Martin Dutertre

**Affiliations:** 1 Institut Curie, PSL Research University, CNRS UMR 3348, F-91405 Orsay, France; 2 Paris Sud University, Paris-Saclay University, CNRS UMR 3348, F-91405 Orsay, France; 3 Equipe Labellisée Ligue Contre le Cancer; 4 Institut Curie Research Center, SIREDO Oncology Center, Paris-Sciences-Lettres Research University, INSERM U830, Laboratory of Biology and Genetics of Cancers, Paris, France; Université Paris Diderot, Sorbonne Paris Cité, France; 5 CNRS UMR 5239, Ecole Normale Supérieure de Lyon, Lyon, France; 6 Unité de Pharmacogénomique, Service de génétique, Institut Curie, Paris, France; Université Paris Descartes, Paris, France; 7 Institut Curie, PSL Research University, Translational Research Department, Experimental Radiotherapy Platform, Orsay, France; 8 Imperial College London, London, UK

## Abstract

Besides analyses of specific alternative splicing (AS) variants, little is known about AS regulatory pathways and programs involved in anticancer drug resistance. Doxorubicin is widely used in breast cancer chemotherapy. Here, we identified 1723 AS events and 41 splicing factors regulated in a breast cancer cell model of acquired resistance to doxorubicin. An RNAi screen on splicing factors identified the little studied ZRANB2 and SYF2, whose depletion partially reversed doxorubicin resistance. By RNAi and RNA-seq in resistant cells, we found that the AS programs controlled by ZRANB2 and SYF2 were enriched in resistance-associated AS events, and converged on the ECT2 splice variant including exon 5 (ECT2-Ex5+). Both ZRANB2 and SYF2 were found associated with ECT2 pre-messenger RNA, and ECT2-Ex5+ isoform depletion reduced doxorubicin resistance. Following doxorubicin treatment, resistant cells accumulated in S phase, which partially depended on ZRANB2, SYF2 and the ECT2-Ex5+ isoform. Finally, doxorubicin combination with an oligonucleotide inhibiting ECT2-Ex5 inclusion reduced doxorubicin-resistant tumor growth in mouse xenografts, and high ECT2-Ex5 inclusion levels were associated with bad prognosis in breast cancer treated with chemotherapy. Altogether, our data identify AS programs controlled by ZRANB2 and SYF2 and converging on ECT2, that participate to breast cancer cell resistance to doxorubicin.

## INTRODUCTION

A major problem in anticancer therapy, either conventional or targeted, is the frequent acquisition of resistance to treatment. One of the main classes of anticancer agents are genotoxic agents. Resistance can involve various processes (often in combination), such as drug efflux or metabolism, drug target regulation, DNA-damage response, cell survival and death pathways, epithelial–mesenchymal transition, and cancer stem cell phenotype (1). Acquired resistance is associated with mutation or expression regulation of genes that are either involved in these processes, or in the expression regulation of such genes. Transcriptomic analyses have found many protein-coding genes, microRNAs and long non-coding RNAs that are differentially expressed in resistant *versus* sensitive cells. While most of these alterations are likely passenger rather than driver events, studies have defined resistance-associated gene regulatory pathways connecting altered regulators and target genes that play a role in resistance. These regulatory pathways have been mainly limited to quantitative gene expression regulation at the levels of transcription, RNA stability, and translation (1,2).

In addition to quantitative regulation, human gene expression is also regulated qualitatively, in a large part through alternative splicing (AS) that generates alternative transcripts in >90% of protein-coding genes. AS is controlled in a large part by >300 splicing factors that bind specific RNA motifs in pre-messenger RNAs (pre-mRNAs) and/or are part of the core spliceosome machinery (3). In various cancers, hundreds of AS regulation events are found in tumors *versus* healthy tissues, and several splicing factors are recurrently mutated or overexpressed in specific cancers and have been shown to have oncogenic properties (4–6). Recent studies on oncogenic splicing factors have started to identify the genome-wide AS programs they control, as well as target splice variants that are phenotypically relevant, suggesting AS regulatory pathways involved in oncogenesis (7–10).

For various anticancer agents, studies on candidate genes have identified splice variants mediating resistance in cellular models or associated with resistance in patients, and a few splicing factors have been involved in resistance (11–14). However, the AS regulatory pathways connecting splicing factors and AS events involved in anticancer drug resistance, are usually unknown. In two studies, the splicing factors PTBP1 and TRA2A were up-regulated in resistant cells and promoted resistance to gemcitabine in pancreatic cancer through AS regulation of the PKM gene, and to paclitaxel in triple-negative breast cancer through AS of RSRC2, respectively (15,16). In addition, very few studies identified genome-wide AS programs in resistant *versus* sensitive cells (17,18), and their role and upstream regulators were not identified. Thus, while AS regulation can play a role in anticancer drug resistance (11–14), AS regulatory pathways and programs involved in anticancer drug resistance remain poorly understood.

To address this question, we studied breast cancer cell resistance to doxorubicin (Doxo), which is commonly used in chemotherapy for this cancer type. AS regulation by Doxo treatment in breast cancer cells has been previously analyzed in the context of acute response (19), but not in the context of resistance. The classical cellular model of acquired Doxo resistance in breast cancer is in the MCF-7 background (20). Here, we identified on a genome-wide level, the sets of AS events and splicing factors regulated at the RNA level in this breast cancer cell model of acquired resistance to doxorubicin, and identified through an siRNA screen two little studied splicing factors (ZRANB2 and SYF2), whose depletion reduced Doxo resistance and subsets of resistance-associated AS events. We found that their genome-wide AS programs converge on the ECT2 splice variant including exon 5 (ECT2-Ex5+), whose depletion reduced Doxo resistance, and that correlated with chemotherapy resistance in breast cancer patients. Thus, this study identifies AS pathways involved in breast cancer cell resistance to Doxo.

## MATERIALS AND METHODS

### Cell culture and treatment

Sensitive (parental MCF-7) and Doxo-resistant (MCF7-DoxoR) human breast carcinoma cells (20) were cultured in 4,5 g/l Glucose DMEM (Eurobio) supplemented with 10% (v/v) fetal bovine serum (Dutscher), and 2 mM l-glutamine (Eurobio) in 5% CO_2_ at 37°C. The resistant phenotype was maintained by culturing MCF7-DoxoR cells in 17 μM Doxo (Sigma). For experiments, Doxo treatments in MCF7-DoxoR cells were done at 150 μM after one passage in the absence of Doxo.

### siRNA transfection

siRNA pools used for the screens were obtained by the cherry-pick library preparation service from Dharmacon. Individual siRNAs were from Eurogentec; sequences are listed in Supplementary Table S1. Reverse transfection was performed at a final concentration of 100 nM with Lipofectamine RNAimax (ThermoFischer Scientific) following the manufacturer's instructions.

### Morpholino oligonucleotide transfection

For RT-PCR and RT-qPCR analysis, MCF7-DoxoR cells were seeded at 300 000 cells/well in a six-well plate. Twenty four hours after seeding, splice-switching oligonucleotide (SSO) or standard control morpholino (Gene Tools, sequences in Supplementary Table S1) were transfected at a final concentration of 5 μM with Endo-Porter PEG (Gene Tools) according to the manufacturer's instruction. Cells were harvested 48 h later for RNA extraction. For WST-1 assay, MCF7-DoxoR cells were seeded at 6000 cells/well in six replicates in a 96-well plate on day 1. Twenty four hours later on day 2, cells were transfected with 5 μM SSO or control morpholino. On day 3, triplicates were treated or not with 150 μM Doxo. Seventy two hours later on day 6, WST-1 assay was performed.

### Plasmid transfection and overexpression analysis

Gateway™ Entry vectors containing ZRANB2 and SYF2 ORF were purchased from the ORFeome Collaboration Collection (Dharmacon) and transferred to Destination vectors adding a C-terminal GFP tag (Vivid Colors™ pcDNA™6.2/EmGFP vector, Invitrogen). 1.5 million parental MCF-7 cells were seeded on a 10 cm dish. Twenty four hours later, 1 μg of CTRL (CAT-GFP), ZRANB2-GFP, or SYF2-GFP vector was transfected with Lipofectamine 2000 (Invitrogen) following manufacturer's instructions. Fourty eight hours after transfection, cells were sorted on FACSAria™ III (BD Bioscences) for GFP+ selection.

### WST-1 cell survival assay

Cells were simultaneously reverse transfected with siRNA and seeded at an appropriate density in 96-well plates (3000, 5000, 4000 and 8000 cells/well for MCF-7, MCF7-DoxoR, MDA-MB-231 and MDA-MB-468 cells, respectively) on day 1, in six replicates per siRNA condition. Forty eight hours later on day 3, triplicates were treated or not with 150 μM Doxo. Seventy two hours later on day 6, cell survival was assayed using WST-1 (Sigma) according to the manufacturer's instructions.

### RNA extraction and RT-PCR

RNA from whole cells was extracted using TRIzol Reagent (ThermoFiscer Scientific), and 1 μl of GlycoBlue (ThermoFisher Scientific) was added for RNA precipitation. Total RNA was treated with DNase I (TURBO DNAfree, ThermoFisher Scientific). Reverse transcription was performed using SuperScript Reverse Transcriptase (ThermoFisher Scientific) and random primers. PCR was performed using GoTaq Flexi DNA Polymerase (Promega), and PCR products were migrated on agarose gels. qPCR was performed using Power SYBR Green PCR Master Mix (ThermoFisher Scientific) on a CFX96 Real-Time PCR Detection System (BioRad). Primer sequences are listed in sequences are listed in Supplementary Table S1.

### Complex capture (2C)

Thirty million MCF7-DoxoR cells were seeded in ten 15 cm dishes, and harvested 48 hours later. Two dishes were used for the non-UV crosslink condition. 2C was performed according to the method described in ref. 21.

### RNA immunoprecipitation

Dynabeads Protein G (ThermoFisher Scientific) were incubated with anti-ZRANB2/ZNF265 antibody (A301-030A, Bethyl Laboratories), anti-SYF2 (16958-1-AP, Proteintech) or normal rabbit IgG (Santa Cruz) at 4°C overnight with rotation. Before cell harvesting, RNA-protein complexes were crosslinked with either 1% formaldehyde (incubation at RT for 10 min, for RIP) or 4000 J/m^2^ ultraviolet radiation (UV Stratalinker 1800, on ice, for CLIP). Formaldehyde crosslink reaction was quenched using 125 mM glycine for 5 min at RT. Cells were washed twice with ice-cold PBS before harvesting and pelleted by centrifugation (4°C, 5 min, 800 g). Cells were resuspended in 1 ml RIPA (Sigma) with Complete Protease Inhibitor Cocktail (Sigma) at 1× concentration and either 1 μl RNAse inhibitor (RNaseOUT, for RIP) or 2 U/ml of RNAse1 (Ambion, for CLIP). Cell lysates were sonicated for 5 min. Supernatant was collected after centrifugation at 4°C for 10 min (10 000 g). An aliquot was used for RNA input and was treated with proteinase K before RNA extraction with TRIzol Reagent (ThermoFisher Scientific). For immunoprecipitation, 400 μg of protein were incubated with antibody-hybridized Dynabeads overnight with rotation at 4°C. Supernatant was removed and beads were washed twice with RIPA buffer before RNA–protein complex elution by incubation with elution buffer (Tris–HCl pH 8 100 mM; Na_2_-EDTA 10 mM; 1% SDS in H_2_O) 3 min at 90°C. Proteins were degraded with proteinase K treatment and RNA was extracted with TRIzol Reagent (ThermoFisher Scientific) for RT-qPCR analysis.

### Western-blot

For immunoblotting, cells were lysed in RIPA buffer (Sigma) after two PBS wash and cell lysate was sonicated for 5 min. Supernatant was collected after centrifugation at 4°C for 10 min (10 000 g). Protein concentration was determined using the Pierce BCA protein assay kit from ThermoFisher Scientific and BSA standards from BioRad. Proteins were then separated in 4–12% NuPAGE Bis–Tris precast gels (ThermoFisher Scientific) and transferred on nitrocellulose membrane using the iBlot2 dry transfer system from ThermoFisher Scientific. Membranes were incubated overnight at +4°C with primary antibodies (anti-ZRANB2: 24816-1-AP, Proteintech; anti-SYF2: 16958-1-AP, Proteintech; anti-GAPDH from Sigma; anti-GFP from Roche) and proteins were detected using horse-radish peroxidase-conjugated goat anti-mouse or anti-rabbit antibodies. After washing, the blots were revealed using Clarity Werstern ECL substrate (BioRad), and a ChemiDoc gel imaging system from BioRad.

### Cell cycle analysis

For cell cycle analysis by FACS with BrdU labelling, BrdU was added to the cells at a concentration of 10 μM, 30 min before or after the 48 h doxorubicin treatment. Cells were trypsinized and fixed by adding 1 ml of cold 70% ethanol and incubating for at least 30 min at −20°C. After centrifugation, cells were resuspended in 0.5% BSA–PBS. Cells were centrifuged again and incubated in 100 μl of 2 M HCl for 20 min at RT. After 20 min, 1 ml of 0.5% BSA–PBS was added and cells were pelleted. After discarding the supernatant, the cell pellet was resuspended in 500 μl of 0.1 M Sodium borate and incubated 2 min at RT. Cells were washed twice with 2 ml of 0.5% BSA–PBS. Cells were then resuspended in 50 μl of 0.5% BSA–PBS with APC-anti BrdU antibody from the APC BrdU Flow kit (BD Bioscience) diluted 1/100, and incubated in the dark at RT for 1 hour. After washing with 1 ml of 0.5% BSA–PBS, cells were incubated in 500 μl of 0.5% BSA–PBS containing of propidium iodide (10 μg/ml) and of RNAse A (0.5 mg/ml) in the dark at RT for 30 min before flow-cytometry analysis. When cells were only labeled with propidium iodide, cells were resuspended in Vindelov solution (Tris–HCl 3,5 mM; NaCl 10 mM; IGEPAL 0.1%) with RNAse A (20 μg/ml) and propidium iodine (50 μg/ml) after fixation, and incubated at RT in the dark for 30 min. Flow-cytometry analysis of 20 000 cells was performed on a FACSCanto flow cytometer from BD Biosciences. Data analysis was done on the FlowJo software.

### Exon-junction array analyses

Three biological replicates were analyzed for each condition. One μg of total RNA was processed and hybridized to Glue Grant Human Transcriptome arrays (Affymetrix) according to the manufacturer's instructions. Data were analyzed with Expression Console (Affymetrix) to perform quality assessment and normalized using quintile normalization. Background correction and probe selection were performed as previously described, using exon annotation from the Faster DB database (19) (http://fasterdb.lyon.unicancer.fr/). Exonic regulation between two conditions was analyzed using the splicing index method in three independent ways, based either on probes in all annotated exons, on probes in annotated alternative exons and their neighbours, or on junction probes. Regulation events with fold change >2 and *P* value < 0.05 were selected. Exonic regulation events were classified into different types (ASE, etc.) based on annotation of known events. Data were visualized in gene context using the Elexir tool of Faster DB.

### RNA-seq analyses

Three biological replicates were analyzed for each condition. Libraries were made using one μg of total RNA and the TruSeq Stranded mRNA Library Preparation Kit (Illumina) involving polyA+ RNA selection. Equimolar pool of libraries were sequenced on a Illumina HiSeq 2500 machine using paired-ends reads (PE, 2 × 101 bp) and High Output run mode allowing to get 200 millions of raw reads per sample. Data were analyzed by two methods. In the first method, raw reads were mapped on the human reference genome hg19 using the STAR aligner (v.2.5.0a). PCR-duplicated reads and low mapping quality reads (MQ < 20) were removed using Picard tools and SAMtools, respectively. Then, rMATS (v3.0.9) (22) was used to identify alternatively skipped exons (ASE), alternative 3′ splice sites (A3SS), alternative 5′ splice sites (A5SS), mutually exclusive exons (MXE) and retained introns (RI) regulated between conditions. Briefly, rMATS uses both spliced reads and exonic reads to calculate percent of spliced-in (PSI) values of alternative exons in replicates, and we used the following thresholds: at least 15 unique reads supporting the splicing event, ΔPSI > 10% (20% for MCF7-DoxoR versus MCF-7), and FDR <0.05. In the second method, RNA-seq data were analyzed using the previously decribed FARLINE algorithm using ΔPSI cutoff as above (23). Results from both methods were pooled. Motif enrichment analysis was performed using the rMAPS software (24). All motif enrichment graphs are available upon request.

### Mouse xenograft experiments

Animal experiments were performed in accordance with the animal welfare and ethical guidelines of Institut Curie. Female Swiss Nu/nu mice were purchased from Charles River Laboratories (L’ARBRESLE, France). Five weeks old mice were injected subcutaneously into the right flank with 10 million MCF7-DoxoR cells in 50 μl Matrigel (Corning) and 50 μl DMEM. When tumors reached 300 mm^3^, mice were randomized into different groups. For tumor growth follow-up, 0.12 mg of vivo-morpholino oligonucleotides VMO-CTRL or VMO-ECT2-Ex5 (Gene Tools, sequences in Supplementary Table S1) in 150 μl PBS were injected intra-tumorally six times every 3 days. On the same day as the third injection, mice received an intraperitoneal injection of either doxorubicin (6 mg/kg) or NaCl solution. Mice were weighed and tumor volume was measured twice a week. For RNA analysis, tumors (four per condition) were injected twice with either VMO-CTRL or VMO-ECT2-Ex5 as above, and 3 days later tumors were removed and used for RNA extraction.

### Patients tumor samples

Tumor collection: Samples of 526 primary unilateral invasive breast tumors excised from women managed at Curie Institute-René Huguenin Hospital (St. Cloud, France) from 1978 to 2008 were analyzed. Immediately after biopsy or surgery, the tumor samples were stored in liquid nitrogen until RNA extraction. All patients (mean age 61.7 years, range 31–91 years) met the following criteria: primary unilateral nonmetastatic breast carcinoma for which complete clinical, histological and biological data were available; no radiotherapy or chemotherapy before surgery; and full follow-up at Curie Institute-René Huguenin Hospital. The patients underwent a physical examination and routine chest radiography every 3 months for 2 years, then annually. Mammograms were done annually. Adjuvant therapy was administered to 369 patients, consisting of chemotherapy (including Doxo or the highly related epirubicin) alone in 91 cases, hormone therapy alone in 176 cases, and both treatments in 102 cases. The histological type and the number of positive axillary nodes were established at the time of surgery. The malignancy of infiltrating carcinomas was scored according to the Scarff-Bloom-Richardson (SBR) histoprognostic grading system. Hormone receptor (HR) [estrogen receptor (ERα), progesterone receptor (PR)] and human epidermal growth factor receptor 2 (ERBB2) status were determined at the protein level by using biochemical methods (dextran-coated charcoal method, enzyme immunoassay, or IHC) and confirmed by qPCR assays. The population was divided into four subtypes: HR+ (ERα+ or PR+)/ERBB2+ (*n* = 58), HR+/ERBB2− (*n* = 294), HR− (ERα− and PR−)/ERBB2+ (*n* = 73) and HR−/ERBB2− (triple-negative subtype, *n* = 101). The median follow-up was 8.9 years (range 130 days to 33.2 years), and 209 patients had a metastasis. Clinicopathological characteristics of patients in relation to metastasis-free survival (MFS) are provided in Supplementary Tables S2 and S3.

### Statistical analyses

Unless otherwise indicated, error bars represent the standard error of the mean, and statistical analyses were done using a *t*-test. *P < 0.05, **P < 0.01, ***P < 0.001. For mouse tumor growth analyses, the Mann–Whitney *U* test was used, and data were computed by using the Graph Pad Prism Software. For patients data, to visualize the efficacy of ECT2 Ex5+/Ex5− isoform ratio and total ECT2 mRNA levels for discriminating between two populations (patients who developed/did not develop metastases) in the absence of an arbitrary cutoff value, data were summarized in a ROC curve. The AUC was calculated as a single measure to discriminate efficacy. Metastasis-free survival (MFS) was determined as the interval between initial diagnosis and detection of the first metastasis. Survival distributions were estimated by the Kaplan–Meier method, and the significance of differences between survival rates were ascertained with the log-rank test. The Cox-proportional hazards regression model was used to assess prognostic significance, and the results are presented as hazard ratios and 95% confidence intervals.

## RESULTS

### Acquired resistance to Doxo is accompanied by widespread AS regulation

**Figure 1.**
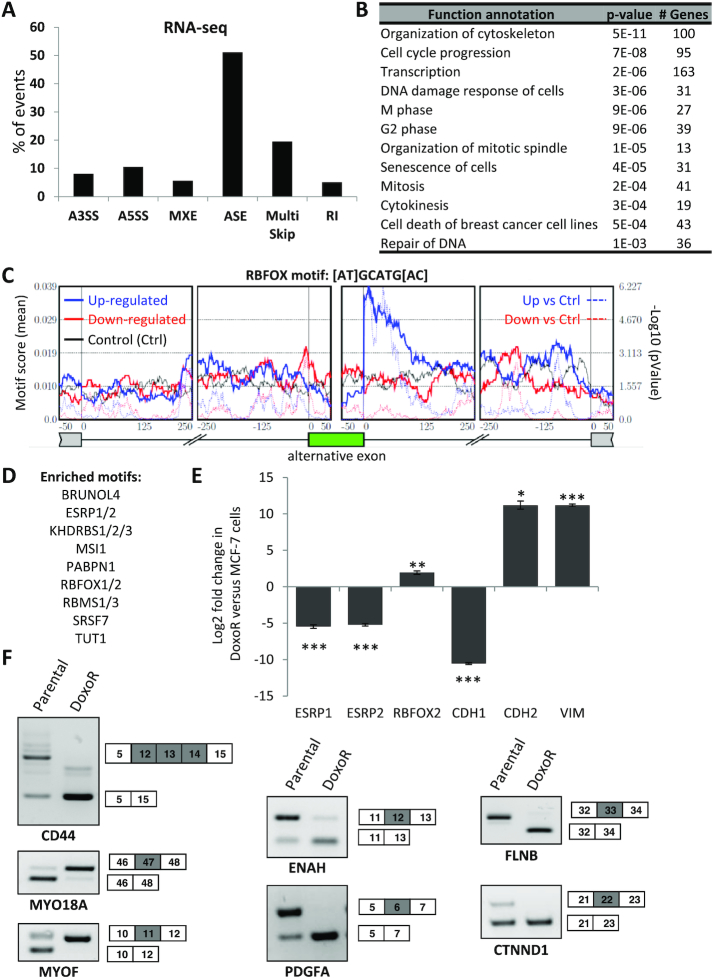
Acquired resistance to Doxo is accompanied by widespread AS regulation. (**A**) Types of AS events regulated in MCF7-DoxoR *versus* MCF-7 cells in RNA-seq data. A3SS, alternative 3′ splice sites. A5SS, alternative 5′ splice sites. ASE, single-exon skipping (cassette exon). RI, retained intron. MXE, mutually exclusive exons. Multi Skip, multiple-exon skipping. (**B**) Enriched functions (Ingenuity Pathway Analysis) in genes with exonic regulation events in RNA-seq data. (**C**) The RBFOX binding motif is enriched downstream of the ASEs (cassette exons) that are upregulated in MCF7-DoxoR *versus* MCF-7 cells. Metagene analysis showing the RBFOX motif enrichment at each nucleotide position around cassette exons. Solid lines represent the motif enrichment score in three lists of exons: up-, down- and non-regulated in MCF7-DoxoR versus MCF-7 cells in our RNA-seq data. Dotted lines represent the *P* value of motif enrichment in regulated exons relative to non-regulated exons. (**D**) Other splicing-related factors, whose binding motif was found to be enriched around ASEs that are regulated in MCF7-DoxoR versus MCF-7 cells. (**E**, **F**) Expression regulation of EMT-related genes and splicing factors (E, RT-qPCR data) and splice variants (F, RT-PCR) between MCF7-DoxoR and MCF-7 cells. RT-qPCR data were normalized to TATA box binding protein (TBP) levels. VIM, vimentin.

To identify on a genome-wide scale, the splice variants regulated in a breast cancer cell model of acquired resistance to Doxo, we used the previously described MCF7-DoxoR cells that are about 200 times less sensitive to Doxo when compared to parental MCF-7 cells (20). We first carried out an exon-junction array analysis, which identified 1271 exonic events, including 706 AS events in 650 genes, that were regulated between MCF7-DoxoR cells and MCF-7 cells (Supplementary Figure S1A and Supplementary Table S4). Later on (while analyzing the impact of splicing factor depletion on alternative exons, see below), we carried out a deeper analysis using RNA-seq, which identified 1723 AS events in 1330 genes regulated between MCF7-DoxoR cells and MCF-7 cells (Figure 1A and Supplementary Table S4). Using both methods, the most prominent type of exonic regulation events were alternatively-skipped exons (ASEs, also called cassette exons), with 37% of regulated ASEs found by array analysis also found by RNA-seq (Supplementary Figure S1B). We also found many regulation events of multiple-exon skipping, alternative 3′ and 5′ splice sites, retained introns, mutually exclusive exons, alternative last exons, and alternative first exons (Figure 1A and Supplementary Figure S1A). Exonic regulation events were enriched in several functions, mainly related to cytoskeleton, transcription, cell cycle, DNA damage response, and cell death (Figure 1B, Supplementary Figure S1C and Supplementary Table S4). There was little overlap with the previously identified set of exons regulated in acute response to Doxo in MCF-7 cells (19) (data not shown). Overall in this study, we validated 30 out of 38 (validation rate of 79%) exonic regulation events tested by RT-PCR (Supplementary Figure S1D and see below).

### An EMT-related splicing switch accompanies, but does not directly explain Doxo resistance

**Figure 2.**
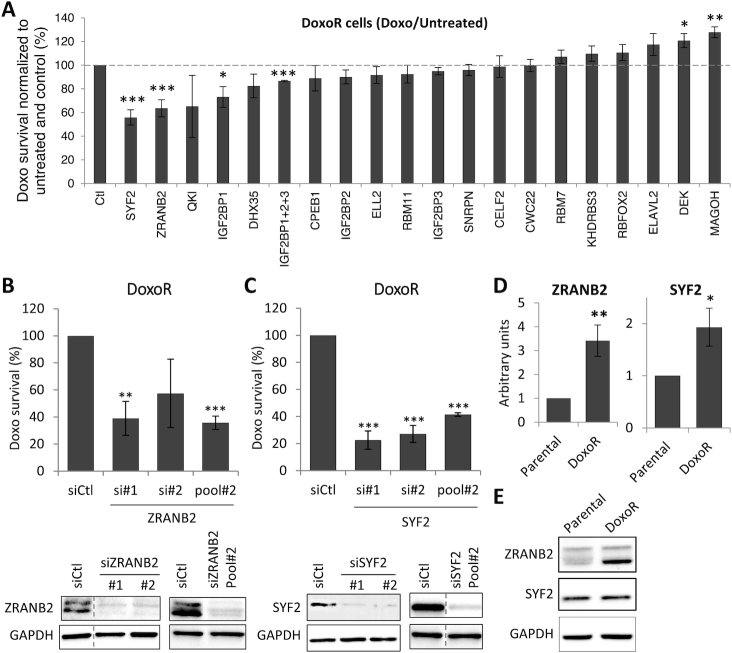
An RNAi screen on splicing factors identifies ZRANB2 and SYF2 as mediators of Doxo resistance. (**A**) RNAi screen in MCF7-DoxoR cells. Following transfection and two-day recovery, cells were grown for three days with or without Doxo, and cell survival was assessed using a WST1 assay. For each siRNA, WST1 signal in the presence of Doxo was divided by WST1 signal in the absence of Doxo, and was expressed as percent of the data obtained with an siRNA that targets no gene (Ctl). (**B**, **C**) Validation of the effects of siRNAs targeting ZRANB2 (B) and SYF2 (C) on their protein levels (Western blot, bottom panels) and Doxo survival (WST1 assay as above, top panels) in MCF7-DoxoR cells. (**D**, **E**) Analysis of ZRANB2 and SYF2 expression levels in MCF7-DoxoR and MCF-7 cells by RT-qPCR (D) and Western blot (E). RT-qPCR data were normalized to 18S RNA levels.

Given the large number of splice variants that were regulated in resistant *versus* sensitive cells, we looked for upstream regulators. For this, we first looked for known RNA motifs corresponding to known RNA-binding proteins and enriched in our RNA-seq dataset of ASEs regulated in resistance. The most enriched motif was an RBFOX binding motif downstream of exons upregulated in MCF7-DoxoR cells (Figure 1C), and other enriched motifs included ESRP1/2 binding motifs (Figure 1D). Interestingly, a switch between RBFOX2 (also called RBM9) and ESRP1/2 splicing factors has been involved in epithelial to mesenchymal transition (EMT) (25), and EMT was previously found associated with Doxo resistance in breast cancer (26–28). Consistently, in comparison to MCF-7 cells, MCF7-DoxoR cells displayed a strong decrease of several epithelial markers including CDH1 and ESRP1/2, and an increase in several mesenchymal markers including CDH2, vimentin and RBFOX2 (Figure 1E). This was accompanied by a splicing switch of several EMT-related splice variants (Figure 1F). Thus, the switch between RBFOX2 and ESRP1/2 likely explains in part the widespread AS regulation events in genes related to cytoskeleton and EMT-related functions that we noted in MCF7-DoxoR *versus* MCF-7 cells (Figure 1B, F). However, depletion of either RBFOX2 in MCF7-DoxoR cells or ESRP1 and 2 (alone or in combination) in MCF-7 cells did not affect Doxo sensitivity in our siRNA screens described below (Figure 2A and Supplementary Figure S2A). While we cannot exclude that depletion of RBFOX2 or ESRP1/2 for longer than 5 days might affect Doxo sensitivity, these data suggest that these factors do not control AS programs directly involved in Doxo sensitivity.

### Identification of the splicing factors ZRANB2 and SYF2 as mediators of Doxo resistance

To further investigate on splicing factors involved in Doxo resistance, we focused on the 41 splicing factors whose expression was either increased or decreased by at least 2-fold in MCF7-DoxoR versus MCF-7 cells in our microarray analysis (Table 1). We then carried out two separate siRNA screens on these two sets of factors, assessing the effect of their depletion on cell survival in the absence and presence of Doxo by using a WST1 assay. The first screen focused on the 22 splicing factors that are more expressed in MCF-7 than MCF7-DoxoR cells, looking for factors, whose depletion in MCF-7 cells would promote doxorubicin resistance. Among these 22 factors, the depletion of only one (SNRPA1) led to a significant increase in MCF-7 cell survival to Doxo when normalized to cell survival in the absence of Doxo; however, this effect was mainly due to a decrease in cell survival in the absence of Doxo (Supplementary Figure S2A and B). The second screen focused on the 19 splicing factors that are more expressed in MCF7-DoxoR cells, looking for factors, whose depletion in these cells would decrease doxorubicin resistance. Among these 19 factors, the depletion of three factors (ZRANB2, SYF2 and IGF2BP1) led to a significant and robust reduction of MCF7-DoxoR cell survival to Doxo when normalized to cell survival in the absence of Doxo (Figure 2A), with little or no impact on cell survival in the absence of Doxo (Supplementary Figure S2C). We did not pursue on IGF2BP1, because its effect on Doxo survival was the least strong, and because there is only little evidence for its function in splicing, as it mainly regulates cytoplasmic mRNA fate and translation (29). Some effect of QKI was seen in some experiments, but it did not reach statistical significance. Further analyses with independent siRNAs confirmed that depletion of either ZRANB2 or SYF2 in MCF7-DoxoR cells decreased their survival to Doxo (Figure 2B and C).

**Table 1.** List of splicing factors regulated in MCF7-DoxoR versus MCF-7 cells

Symbol	Description	Regul	Fold change
IGF2BP2	Insulin-like growth factor 2 mRNA binding protein 2	Up	17.8
IGF2BP3	Insulin-like growth factor 2 mRNA binding protein 3	Up	14.1
SNRPN	Small nuclear ribonucleoprotein polypeptide N	Up	13.7
IGF2BP1	Insulin-like growth factor 2 mRNA binding protein 1	Up	12.3
RBM7	RNA binding motif protein 7	Up	4.9
CPEB1	Cytoplasmic polyadenylation element binding protein 1	Up	4.8
ELL2	Elongation factor, RNA polymerase II, 2	Up	3.5
KHDRBS3	KH domain containing, RNA binding, signal transduction associated 3	Up	3.5
DHX35	DEAH (Asp-Glu-Ala-His) box polypeptide 35	Up	3.1
ZRANB2	Zinc finger, RAN-binding domain containing 2	Up	3.1
CWC22	CWC22 spliceosome-associated protein homolog (*S. cerevisiae*)	Up	3.0
QKI	Quaking homolog, KH domain RNA binding (mouse)	Up	2.8
RBM11	RNA binding motif protein 11	Up	2.8
SYF2	SYF2 homolog, RNA splicing factor (*S. cerevisiae*)	Up	2.7
ELAVL2	ELAV (embryonic lethal, abnormal vision, Drosophila)-like 2 (Hu antigen B)	Up	2.5
RBM9	RNA binding motif protein 9 (RBFOX2)	Up	2.4
MAGOH	Mago-nashi homolog, proliferation-associated (Drosophila)	Up	2.4
CELF2	CUGBP, Elav-like family member 2	Up	2.3
DEK	DEK oncogene	Up	2.1
RBM47	RNA binding motif protein 47	Down	11.0
ESRP2	Epithelial splicing regulatory protein 2	Down	10.4
RBPMS	RNA binding protein with multiple splicing	Down	8.0
BCAS1	Breast carcinoma amplified sequence 1	Down	6.8
TTF2	Transcription termination factor, RNA polymerase II	Down	5.4
JUP	Junction plakoglobin	Down	5.2
RBM23	RNA binding motif protein 23	Down	5.2
TRIM24	Tripartite motif-containing 24	Down	4.7
LEO1	Leo1, Paf1/RNA polymerase II complex component, homolog (S. cerevisiae)	Down	4.4
RBM24	RNA binding motif protein 24	Down	4.3
SRPK2	SRSF protein kinase 2	Down	3.5
PCBP3	Poly(rC) binding protein 3	Down	3.2
POLR2J	Polymerase (RNA) II (DNA directed) polypeptide J, 13.3kDa	Down	3.2
ZFP36	Zinc finger protein 36, C3H type, homolog (mouse)	Down	3.0
ZFP36L2	Zinc finger protein 36, C3H type-like 2	Down	3.0
BCAS2	Breast carcinoma amplified sequence 2	Down	3.0
SRRT	Serrate RNA effector molecule homolog (Arabidopsis)	Down	2.9
SNRPA1	Small nuclear ribonucleoprotein polypeptide A'	Down	2.7
HNRNPAB	Heterogeneous nuclear ribonucleoprotein A/B	Down	2.5
ESRP1	Epithelial splicing regulatory protein 1	Down	2.4
HNRNPUL1	Heterogeneous nuclear ribonucleoprotein U-like 1	Down	2.2
PRPF4	PRP4 pre-mRNA processing factor 4 homolog (yeast)	Down	2.0

In addition, RT-qPCR analyses confirmed our microarray data indicating overexpression of both the *ZRANB2* and *SYF2* genes in MCF7-DoxoR cells compared to MCF-7 cells (Figure 2D). At the protein level, Western blot analysis showed that ZRANB2 was overexpressed in MCF7-DoxoR cells compared to MCF-7 cells (Figure 2E). We detected two bands, both of which were depleted by ZRANB2 siRNAs, but only one of which was overexpressed in MCF7-DoxoR cells (Figure 2B,E). Global SYF2 protein levels were not higher in MCF7-DoxoR cells than in MCF-7 cells (Figure 2E), but we cannot rule out cell heterogeneity of its expression levels. These data indicate that both ZRANB2 and SYF2 are upregulated at the global mRNA level in MCF7-DoxoR *versus* parental MCF-7 cells, and suggest that they are also regulated at other (post-transcriptional or post-translational) levels in this model.

### Identification of exons regulated by ZRANB2

**Figure 3.**
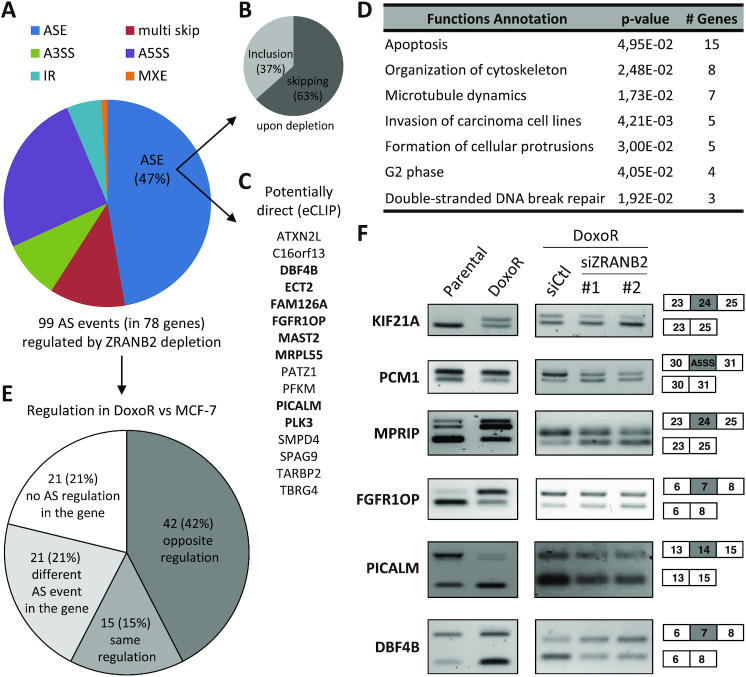
Identification of exons regulated by ZRANB2. (**A**) Types of AS events regulated by ZRANB2 depletion for 48 h in MCF7-DoxoR cells (RNA-seq analysis). (**B**) Direction of regulation of ASE events by ZRANB2 depletion. (**C**) ZRANB2-regulated ASEs with a nearby ZRANB2-binding site found by CLIP-seq. In bold, events that were validated by RT-PCR for regulation by ZRANB2 (all the tested events were validated). (**D**) Enriched functions (Ingenuity Pathway Analysis) in genes with AS regulation by ZRANB2. (**E**) Comparison of ZRANB2-regulated AS events with AS events regulated in MCF7-DoxoR versus MCF-7 cells. (**F**) RT-PCR validations of AS events regulated by siRNAs targeting ZRANB2 (whose depletion was verified in Figure 2B) and in MCF7-DoxoR *versus* MCF-7 cells. The effects of ZRANB2 siRNAs on FGFR1OP were weak but reproducible.

ZRANB2 is well known to bind RNA, and was shown to regulate AS of several genes (30), but little is known about its biological function and its AS targets genome-wide. To identify the AS events it regulates in MCF7-DoxoR cells, we analyzed by RNA-seq the effects of its depletion using two independent siRNAs, and three biological replicates for each. Focusing on genes that were regulated at the AS level by both siRNAs, we identified 99 regulated exons in 78 genes (Supplementary Figure S3A and Supplementary Table S5). The main type of AS event regulated by ZRANB2 was ASEs (47%; Figure 3A), with ZRANB2 depletion inducing skipping in 63% cases (Figure 3B). We validated 15 out of 22 events tested by RT-PCR (validation rate of 68%; Supplementary Figure S3B and see below). In addition, 16 (31%) ASE exons regulated by ZRANB2 had a nearby ZRANB2 binding site in a CLIP-seq dataset available from ENCODE (31), suggesting direct regulation (Figure 3C). The 99 AS events regulated by ZRANB2 were enriched in genes involved in apoptosis, microtubule dynamics, cell cycle and DNA repair (Figure 3D). Remarkably, 55 (71%) of the 78 genes with AS regulation by ZRANB2 also had AS regulation in resistance. More precisely, 42 (42%) of the 99 AS events regulated by ZRANB2 depletion were regulated in the opposite direction in MCF7-DoxoR versus MCF-7 cells, while only 15 events (15%) were regulated in the same direction (Figure 3E). This bias is consistent with ZRANB2 being overexpressed in MCF7-DoxoR cells and promoting resistance, and we validated several opposite regulation events by RT-PCR (Figure 3F and see below, Figure 5).

### The core spliceosome component SYF2 controls AS, mainly alternative 3′ splice sites, in specific genes

**Figure 4.**
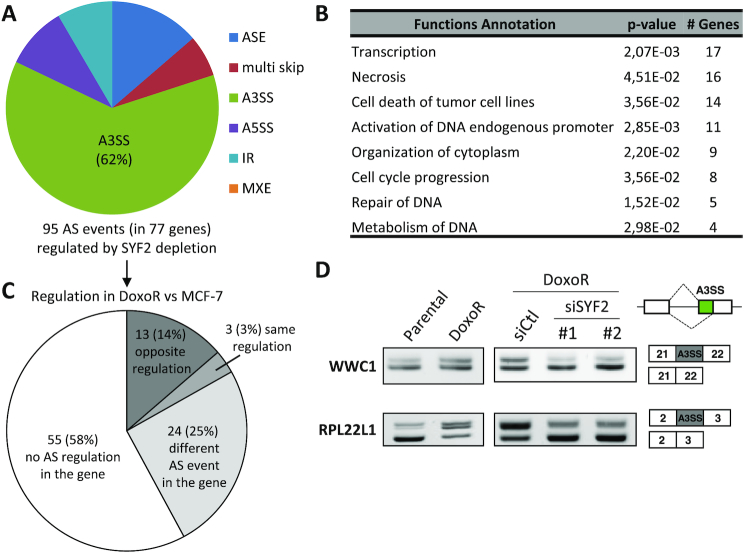
The core spliceosome component SYF2 controls AS of specific genes. (**A**) SYF2 depletion for 48 h in MCF7-DoxoR cells mainly regulates alternative 3′ splice sites (RNA-seq analysis). (**B**) Enriched functions (Ingenuity Pathway Analysis) in genes with AS regulation by SYF2. (**C**) Comparison of SYF2-regulated AS events with AS events regulated in MCF7-DoxoR versus MCF-7 cells. (**D**) RT-PCR validations of A3SS events regulated by siRNAs targeting SYF2 (whose depletion was verified in Figure 2C) and in MCF7-DoxoR versus MCF-7 cells.

**Figure 5.**
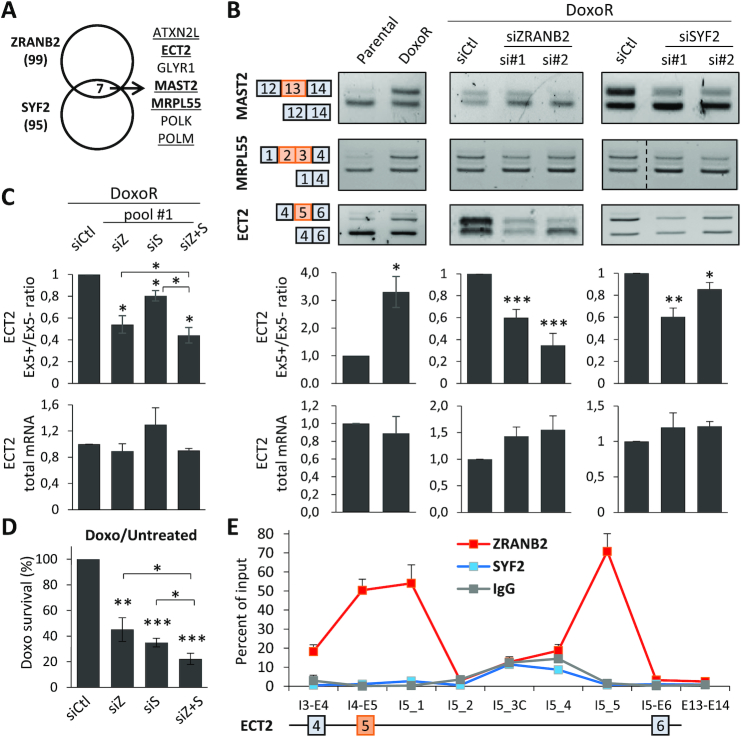
ZRANB2 and SYF2 splicing programs converge on the ECT2-Ex5+ splice variant. (**A**) Overlap between AS events regulated by ZRANB2 and SYF2 in RNA-seq. Events that were regulated in the opposite direction in MCF7-DoxoR versus MCF-7 cells are underlined, and those that were validated by RT-PCR are in bold. (**B**) RT-PCR validation of AS regulation in the *MAST2*, *MRPL55* and *ECT2* genes and RT-qPCR analysis of ECT2 isoforms and total mRNA levels in MCF7-DoxoR *versus* MCF-7 cells, and in MCF7-DoxoR cells transfected with siRNAs targeting ZRANB2 and SYF2 (whose depletion was verified in Figure 2B and C). (**C**, **D**) RT-qPCR analysis of ECT2 isoforms and total mRNA levels (C), and WST1 analysis of Doxo survival (D, normalized to untreated cells as in Figure 2) in MCF7-DoxoR cells transfected with siRNA pools #1 targeting ZRANB2 (siZ) and SYF2 (siS) alone and in combination (siZ+S). ZRANB2 and SYF2 deletion are shown in Supplementary Figure S4F. (**E**) CLIP-qPCR analysis of ZRANB2 and SYF2 association with ECT2 pre-mRNA at the indicated locations. IgG, control immunoglobulin.

SYF2 is a component of the core spliceosome (32). To identify the AS events regulated by SYF2 in MCF7-DoxoR cells, we analyzed by RNA-seq the effects of its depletion using two independent siRNAs and three biological replicates for each. Focusing on genes that were regulated at the AS level by both siRNAs, we identified 95 regulated exons in 77 genes (Supplementary Figure S3C and Supplementary Table S6). The main type of AS event regulated by SYF2 was A3SS (62%; Figure 4A), which is in sharp contrast with ZRANB2 (9% A3SS; Figure 3A) and with most splicing factors studied so far. We validated 14 out of 21 events tested by RT-PCR (validation rate of 67%), including many A3SS events (Supplementary Figure S3D and see below). The AS events regulated by SYF2 were enriched in genes involved in transcription, cell death, cell cycle and DNA repair (Figure 4B). We found that 33 (43%) of the 77 genes with AS regulation by SYF2 depletion also had AS regulation in MCF7-DoxoR versus MCF-7 cells, and 13 (14%) of the 95 AS events regulated by SYF2 depletion were regulated in the opposite direction in MCF7-DoxoR versus MCF-7 cells (Figure 4C). This 14% overlap is lower than the 42% overlap that we observed in the case of ZRANB2 (Figure 3E), and this is consistent with the less robust overexpression of SYF2 compared to ZRANB2 in MCF7-DoxoR cells (Figure 2D-E). Meanwhile, only three (3%) AS events were regulated in the same direction (Figure 4C). The higher proportion of regulation events in the opposite (14%) rather than same direction (3%), is consistent with SYF2 promoting resistance, and we validated several opposite regulation events by RT-PCR (Figure 4D and see below, Figure 5).

### ZRANB2 and SYF2 splicing programs converge on the ECT2-Ex5+ splice variant

We then investigated, whether the shared ability of ZRANB2 and SYF2 to promote Doxo resistance may be mediated by common splicing targets. Although they mainly regulated different types of AS events, as noted above in our RNA-seq analyses (Figures 3A and 4A), we found seven AS events, that were similarly regulated by both ZRANB2 and SYF2, including five that were regulated in the opposite direction in MCF7-DoxoR versus MCF-7 cells (underlined in Figure 5A). For two of them (*ATXN2L* and *POLM* genes), multiple AS events were detected by RT-PCR, thus complicating their validation (Supplementary Figure S4A and B). For the *ECT2*, *MAST2* and *MRPL55* genes, we validated by RT-PCR that the same AS event was regulated in a similar manner by both ZRANB2 and SYF2 depletion in MCF7-DoxoR cells, and in the opposite direction in MCF7-DoxoR versus MCF-7 cells (Figure 5B). In all three cases, exon inclusion was higher in MCF7-DoxoR versus MCF-7 cells, and was decreased by both ZRANB2 and SYF2 depletion in MCF7-DoxoR cells and in two additional breast cancer cell lines (Figure 5B and Supplementary Figure S4C). In the case of MRPL55, the AS event involves two successive cassette exons (exons 2 and 3). In the case of MAST2, the regulated exon (exon 13) is a microexon of 21 nucleotides. The case of *ECT2* involves a more usual single cassette exon, and we therefore focused on it for further studies.

Detailed RT-qPCR analyses showed that ECT2 exon 5 (ECT2-Ex5) inclusion level was higher in MCF7-DoxoR than in MCF-7 cells, and was reduced by depletion of either ZRANB2 or SYF2 with various siRNAs, while total ECT2 mRNA levels were not significantly regulated (Figure 5B, C and Supplementary Figure S4D). Overexpression of either ZRANB2 or SYF2 in MCF-7 cells did not alter the ECT2-Ex5+/ECT2-Ex5- ratio (Supplementary Figure S4E); this could be due either to the added tag, to the nature of the transfected isoforms, or to the lack of appropriate cofactors (or upstream regulators) of SYF2 and ZRANB2 in parental MCF-7 cells. Combined depletion of both ZRANB2 and SYF2 had additive, but not synergistic effects on both ECT2 Ex5 inclusion and Doxo survival (Figure 5C, D and Supplementary Figure S4F), suggesting no interaction between these two factors despite their convergence on ECT2 Ex5 splicing. Altogether, our data demonstrate that both ZRANB2 and SYF2 promote ECT2-Ex5 inclusion in MCF7-DoxoR cells, and suggest that the high levels of ZRANB2 in these cells likely contribute to high ECT2-Ex5 inclusion levels, although we cannot exclude that other factors might also contribute.

### ZRANB2 and SYF2 associate with ECT2 pre-mRNA

To determine, whether the regulation of ECT2-Ex5 inclusion by ZRANB2 and SYF2 may be direct, we analyzed the association of these two factors with ECT2 pre-mRNA by CLIP-qPCR, which involves *in cellulo* crosslinking of direct RNA-protein interactions by ultraviolet irradiation. We carried out qPCR at various locations from exons 4 to 6 of ECT2 pre-mRNA, so as to identify binding sites that could explain regulation of alternative exon 5. These CLIP-qPCR analyses found robust binding of ZRANB2 at specific locations in ECT2 pre-mRNA, namely near exon 5 and upstream the 3′ end of intron 5 (Figure 5E). Both binding regions coincide with sequences resembling the known consensus ZRANB2-binding bipartite RNA motif (two closely spaced AGGUAA motifs (33); Supplementary Figure S11). We also found ZRANB2 binding near the regulated exon 13 in MAST2 pre-mRNA (Supplementary Figure S5D). In both ECT2 and MAST2, a potential ZRANB2-binding bipartite RNA motif overlaps the 5′ splice of the alternative exon, whose inclusion is promoted by ZRANB2 (Supplementary Figure S11 and S12). Altogether, these data suggest that the regulation of ECT2-Ex5 and MAST2-Ex13 inclusion by ZRANB2 may involve direct binding in their close vicinity. However, we cannot exclude a potential role for additional binding sites located farther in the flanking introns, as detected in ECT2 by CLIP-qPCR (Figure 5E), and in both genes in the above-mentioned CLIP-seq dataset (Supplementary Figure S5A-B).

Regarding SYF2, by CLIP-qPCR we did not detect it at any location between ECT2 exons 4 and 6 (Figure 5E). However, it is so far unknown, whether SYF2 can bind RNA directly. Thus, our negative CLIP-qPCR data could be due either to a lack of SYF2 binding to ECT2 pre-mRNA, or to technical failure. To determine, whether SYF2 can bind RNA, we used the recently described complex-capture (2C) method, involving *in cellulo* crosslinking of direct RNA-protein interactions by ultraviolet irradiation, followed by cell lysis, nucleic acid purification on a matrix-based column, and Western blot analysis (21). Using 2C on MCF7-DoxoR cells, we detected both SYF2 and ZRANB2 proteins (but not histone H3) associated to nucleic acids; signal was dependent on UV crosslink and was abolished by RNase treatment, thus indicating that SYF2 binds cellular RNA (Supplementary Figure S5C). Then, to verify that immunoprecipitated SYF2 can be found indirectly associated with ECT2 pre-mRNA, as expected for a component of the core spliceosome (32), we carried out RIP-qPCR, where both RNA-protein and protein-protein interactions are crosslinked with formaldehyde. By RIP-qPCR, we detected a significant association of SYF2 with ECT2 pre-mRNA, using primers around each intron-exon boundary (including at exon 5), with less signal at the beginning of intron 5 (Supplementary Figure S5E). This pattern is consistent with SYF2 being in the core spliceosome, and with our finding that it mainly regulates 3′ splice sites (Figure 4A). Altogether, our data suggest that SYF2 associates with ECT2 pre-mRNA, most likely not through direct RNA binding (although it can bind RNA) but as part of the spliceosome. In conclusion, our data suggest that the regulation of ECT2-Ex5 inclusion by ZRANB2, and probably SYF2, is direct.

### ZRANB2, SYF2 and the ECT2-Ex5+ isoform promote Doxo resistance and S phase accumulation

**Figure 6.**
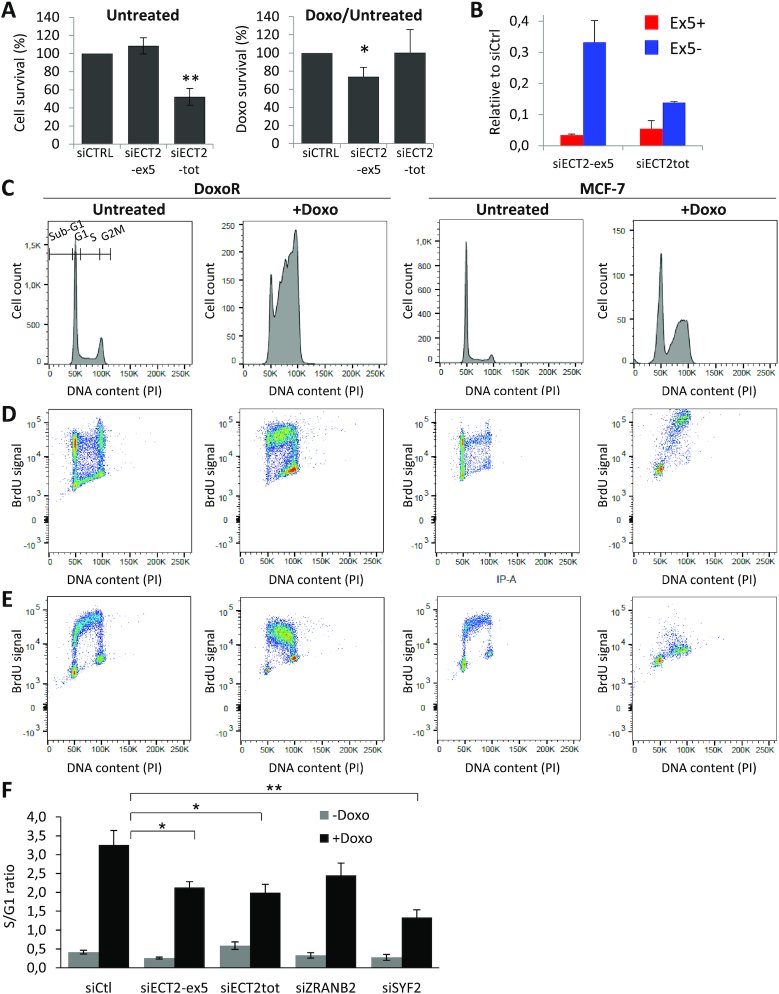
The ECT2-Ex5+ splice variant promotes Doxo resistance and S phase accumulation. (**A**) Effects of siRNAs targeting either exon 5 (siECT2-ex5) or constitutive exons of ECT2 (siECT2tot) on MCF7-DoxoR cell survival in the absence and presence of Doxo 150 μM, as measured by WST1 assay and expressed as percent of control siRNA, as in Figure 2. (**B**) RT-qPCR analysis of ECT2 Ex5+ and Ex5- isoforms. (**C–E**) Cell cycle analysis by FACS in MCF7-DoxoR and MCF-7 cells treated for 48 hours with Doxo at 150 and 0.5 μM, respectively. DNA content was measured with propidium iodide (PI). Replicating DNA was pulse-labeled with BrdU either at the beginning (D) or at the end (E) of a 48-hour incubation with or without Doxo. (**F**) Effects of ZRANB2, SYF2 and ECT2-Ex5+ depletion on MCF7-DoxoR cell cycle in the absence and presence of Doxo. The proportion of cells in G1 and S phase was measured by FACS with PI staining.

To determine, whether the ECT2 isoform including exon 5 (ECT2-Ex5+) may play a role in Doxo resistance, we then transfected MCF7-DoxoR cells with siRNAs targeting different exons of ECT2. Depletion of total ECT2 using an siRNA pool targeting constitutive exons (siECT2-tot) strongly decreased MCF7-DoxoR cell survival in the absence of Doxo, and did not decrease cell survival to Doxo 150 μM normalized to untreated cells (Figure 6A, B and Supplementary Figure S6A). In contrast, depletion of the ECT2-Ex5+ isoform using an siRNA pool targeting exon 5 (siECT2-ex5) had no impact on MCF7-DoxoR cell survival in the absence of Doxo, but reduced by 26% their survival to Doxo normalized to untreated cells (Figure 6A-B and Supplementary Figure S6A). This effect represents between one half and one third of the effects of SYF2 and ZRANB2 depletion (comparing Figure 6A and Figure 2B, C). These data suggest that the ECT2-Ex5+ isoform plays a role specifically in cell survival to Doxo. In addition, because isoform depletion by siRNA leads to decreased gene expression levels, we also used a splice-switching oligonucleotide (SSO-ECT2-Ex5) that targets the 5′ splice site of ECT2 exon 5 and induces its skipping without affecting total ECT2 mRNA levels (Supplementary Figure S6A-B). SSO-ECT2-Ex5 significantly decreased MCF7-DoxoR cell survival to Doxo without affecting their survival in the absence of the drug (Supplementary Figure S6C). These data demonstrate the role of the ECT2-Ex5+ isoform in Doxo survival. We also tested the effects of siRNAs targeting 5 other exons regulated in MCF7-DoxoR *versus* MCF-7 cells and by either ZRANB2 or SYF2; only the siRNA targeting ECT2-Ex5 significantly affected Doxo survival (Supplementary Figure S6D), thus further showing the specificity of its effects.

To better understand the resistance phenotype of MCF7-DoxoR cells, we monitored cell cycle progression of parental MCF-7 and resistant MCF7-DoxoR cells following a 48-hour treatment with their respective IC50 dose of Doxo (i.e. 150 μM for MCF7-DoxoR cells and 0.5 μM for MCF-7 cells). In MCF7-DoxoR cells, Doxo induced a striking decrease of the proportion of cells in G1 phase that mainly resulted from a massive accumulation of cells in S phase and a modest increase in G2-M (Figure 6C and Supplementary Figure S7A). In comparison, in parental MCF-7 cells, Doxo induced less accumulation of cells in S phase and induced more cells in sub-G1 (cell death; Figure 6C and data not shown). To further analyze the effects of Doxo on cell cycle progression, DNA-replicating cells were pulse-labeled with BrdU at the beginning of a 48-hour incubation with or without Doxo. In the case of untreated cells (both MCF7-DoxoR and MCF-7), BrdU^+^ cells were able to progress through G2/M and G1 phase within 48 hours (Figure 6D). In the case of Doxo treatment, MCF7-DoxoR cells showed a strong accumulation of BrdU^+^ cells in early, mid and late S phase, while BrdU^+^ MCF-7 cells mainly accumulated in late S and G2/M (Figure 6D). These results suggest that in response to Doxo, MCF7-DoxoR cells may have a stronger ability (when compared to MCF-7 cells) to either slow down or inhibit DNA replication in early and mid S phase. To further analyze S phase progression and replication activity, we also performed BrdU labeling at the end of the 48-hour incubation with or without Doxo. We observed in untreated MCF7-DoxoR and MCF-7 cells the ‘horse shoe-like’ shape showing BrdU^+^ early, mid and late S-phase cells, and BrdU-negative G1 and G2/M cells. In the case of Doxo treatment, MCF7-DoxoR cells showed a massive accumulation of BrdU^+^ cells in S phase and a significant reduction of BrdU^−^ cells in G1, indicating that most MCF7-DoxoR cells were replicating DNA following a 48-hour Doxo treatment (Figure 6E). In contrast, in the case of Doxo-treated MCF-7 cells, we observed a strong reduction of BrdU^+^ cells in S phase and a strong accumulation of BrdU^−^ cells in late S and G2/M, indicating that most MCF-7 cells were unable to replicate DNA following a 48-hour Doxo treatment (Figure 6E). Collectively, these results suggest that a 48-hour Doxo treatment induces a slowdown of DNA replication in MCF7-DoxoR cells (they are still able to replicate DNA, but slowly), whereas it prevents DNA replication in MCF-7 cells (they cannot replicate DNA). These data suggest that the resistance phenotype of MCF7-DoxoR cells may be linked to their ability to slow down S phase progression without preventing DNA replication in response to high doses of Doxo.

Depletion of either ZRANB2, SYF2 or the ECT2-Ex5+ isoform in MCF7-DoxoR cells had little effect on cell cycle in the absence of Doxo and on cell death (sub-G1) in the absence or presence of Doxo, but reduced the accumulation of cells in S phase in response to Doxo (Figure 6F and Supplementary Figure S7B). Depletion of total ECT2 also reduced the S to G1 ratio in the presence of Doxo (Figure 6F), but this effect is difficult to interpret, because depletion of total ECT2 strongly increased cell death and polyploidy in the absence of Doxo (Supplementary Figure S7B). These data indicate that ZRANB2, SYF2 and the ECT2-Ex5+ isoform contribute to the Doxo-induced S-phase accumulation phenotype of resistant cells.

### High ECT2-Ex5 inclusion levels promote tumor growth of Doxo-resistant cells, and are associated with chemotherapy resistance in breast cancer patients

**Figure 7.**
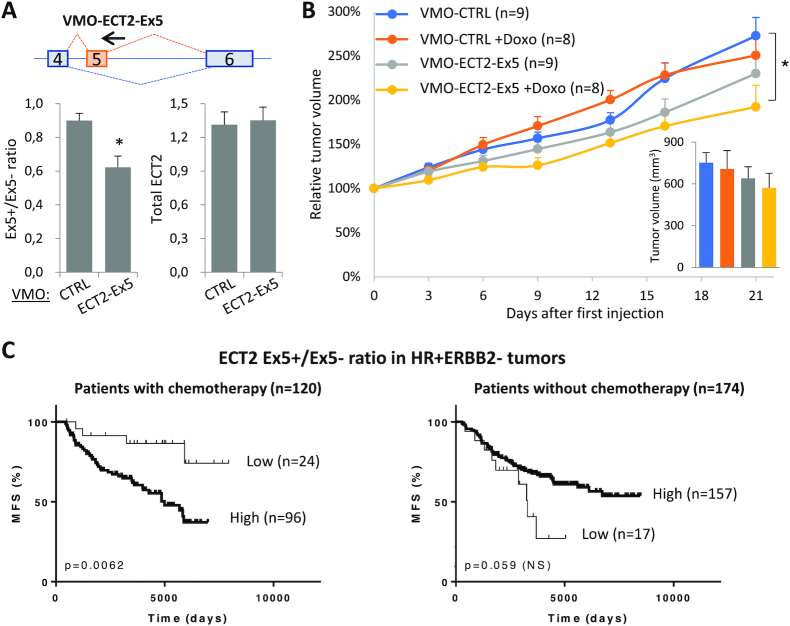
High ECT2-Ex5 inclusion levels promote tumor growth of MCF7-DoxoR cells, and are associated with chemotherapy resistance in breast cancer patients. (**A**) RT-qPCR analysis of ECT2 isoforms ratio and total ECT2 mRNA levels in MCF7-DoxoR tumors injected with the indicated VMOs. (**B**) Mean relative volume of tumors injected with the indicated VMOs and with or without Doxo treatment. Insert, absolute tumor volume at day 21. (**C**) High inclusion levels of ECT2-Ex5 measured in HR+ ERBB2– breast tumors before treatment, correlate with bad prognosis in patients that were treated with chemotherapy. Kaplan-Meier curves. MFS, metastasis-free survival.

We then investigated on the function of ECT2 splicing isoforms in tumor growth of Doxo-resistant cells. For this, MCF7-DoxoR cells were implanted in mice, where they formed tumors. Then, tumors were injected either with a vivo-morpholino oligonucleotide (VMO) that targets the 5′ splice site of ECT2 exon 5 to inhibit its inclusion (VMO-ECT2-Ex5, Figure 7A) or with a negative-control VMO (VMO-CTRL). RT-qPCR analysis on tumors showed that the ECT2 Ex5+/ Ex5- isoform ratio was significantly, but partially reduced by VMO-ECT2-Ex5 when compared to VMO-CTRL with no effect on total ECT2 mRNA levels (Figure 7A). In further experiments, tumors injected with either VMO-ECT2-Ex5 or VMO-CTRL were treated or not with the maximal tolerable dose of Doxo (6 mg/kg), and tumor growth was followed in 8 or 9 animals per group by measuring tumor volume twice a week (mice started to die from Doxo toxicity at day 21, thus limiting tumor follow-up). Doxo treatment did not inhibit the growth of VMO-CTRL tumors (*P* = 0.49; Figure 7B), which is consistent with the high resistance of MCF7-DoxoR cells. In the absence of Doxo treatment, VMO-ECT2-Ex5 tumors tended to grow less than VMO-CTRL tumors (*P* = 0.25; Figure 7B). Interestingly, the combination of VMO-ECT2-Ex5 and Doxo treatment significantly decreased tumor growth (*P* = 0.0152; comparing VMO-ECT2 + Doxo with VMO-CTRL; Figure 7B). Finally, when pooling data from Doxo-treated and untreated mice, VMO-ECT2-Ex5 tumors were growing less than VMO-CTRL tumors (*P* = 0.036). The small amplitude of VMO-ECT2-Ex5 effects may be due to its moderate efficacy in decreasing ECT2-Ex5 inclusion levels (Figure 7A). Thus, our data suggest that higher inclusion levels of ECT2-Ex5 promote tumor growth of MCF7-DoxoR cells, and indicate that the growth of Doxo-resistant tumors can be inhibited by combined Doxo and VMO-ECT2-Ex5 treatments, although it is unclear for statistical reasons, whether VMO-ECT2-Ex5 may decrease MCF7-DoxoR tumor growth preferentially in the presence of Doxo.

To determine whether the alternative inclusion of ECT2-Ex5 may have relevance to chemotherapy resistance in breast cancer patients, we quantitated ECT2 Ex5+ and Ex5- isoforms (as well as total ECT2 mRNA) by RT-qPCR in a collection of 526 clinically annotated breast tumors that were collected before they were treated with or without chemotherapy, which in 95% cases used Doxo or the highly related eprirubicin (Supplementary Table S2). Overall, when combining the different subtypes of breast cancer, high levels of ECT2-Ex5 inclusion (ECT2 Ex5+ / ECT2 Ex5- ratio) were associated with shorter metastasis-free survival in patients that were treated with chemotherapy, but not in patients that were not treated with chemotherapy (Supplementary Figure S8A). This association of high levels of ECT2-Ex5 inclusion with bad prognosis specifically in case of chemotherapeutic treatment, was even more pronounced in the HR+ERBB2– subtype of breast cancer (Figure 7C). In contrast, high levels of ECT2-Ex5 inclusion were not associated with bad prognosis in triple-negative breast cancer patients treated with chemotherapy (Supplementary Figure S8B). Consistently, we found that depletion of the ECT2-Ex5+ isoform (as well as depletion of ZRANB2 or SYF2) did not affect Doxo survival in two triple-negative breast cancer cell lines (Supplementary Figure S9). Similarly to high levels of ECT2-Ex5 inclusion, high levels of total ECT2 mRNA were associated with bad prognosis in HR+ERBB2- patients treated with chemotherapy (Supplementary Figure S8C, left panel). However, in this case, a similar trend towards significance (*P* = 0.052) was seen in patients that were not treated with chemotherapy (Supplementary Figure S8C, right panel), in contrast with high levels of ECT2-Ex5 inclusion that tended to be of good prognosis in patients without chemotherapy (Figure 7C, right panel). Finally, multivariate analysis indicated that high inclusion levels of ECT2-Ex5 were an independent predictive marker of bad prognosis in the subgroup of 120 HR+ERBB2- breast cancer patients treated with chemotherapy (Supplementary Tables S3 and S7). Altogether, these data indicate that high inclusion levels of ECT2-Ex5 are a predictive marker of bad prognosis in breast cancer, specifically in the HR+ERBB2- subtype (as opposed to triple-negative tumors) and in patients that were treated with chemotherapy. These data are highly consistent with our *in vitro* findings described above, that the depletion of the ECT2-Ex5+ isoform (but not the depletion of total ECT2 mRNA) decreased the survival of MCF7-DoxoR cells specifically in the presence of Doxo (Figure 6A and Supplementary Figure S6C), and did not decrease Doxo survival of triple-negative cell lines (Supplementary Figure S9).

## DISCUSSION

Recent studies have started to identify AS networks involved in oncogenesis, but little is known about AS regulatory pathways and genome-wide programs involved in resistance to anticancer treatments. In this study, we identified genome-wide AS programs associated with, as well as AS regulatory pathways controlling breast cancer cell resistance to Doxo. These programs and pathways involve two little studied splicing factors (ZRANB2 and SYF2), and converge on an AS event (ECT2-Ex5 inclusion) that we characterize functionally.

*ECT2*, also known as *ARHGEF31*, is a protooncogene with a well established role in the cytokinesis phase of the cell cycle (34). Our data suggest that the ECT2-Ex5+ isoform promotes Doxo-induced S-phase accumulation and Doxo resistance. Interestingly, the exon 5 of ECT2 encodes a BRCT (BRCA1 carboxy-terminal) domain, which is often found in proteins involved in the DNA damage response (35), and ECT2 was recently involved in cell death induction by DNA damage and in cell sensitivity to an inhibitor of ATR, a key mediator of replication stress response (36–38). Further experiments will be required to test the role of the BRCT domain encoded by ECT2 exon 5, which is complicated by the fact that it is adjacent to two other BRCT domains in the protein (35). Interestingly, a splice variant of ECT2 was recently found to be regulated by paclitaxel in lung cancer cells, and to mediate inhibition of cell proliferation (39).

Our data show a role of ZRANB2 and SYF2 in Doxo resistance. ZRANB2, also known as ZIS or ZNF265, is a ubiquitous RNA-binding protein that was shown to interact with several splicing factors and a specific RNA motif, and to regulate AS of several minigenes, but little was known about its endogenous splicing targets and its biological functions (30,33,40). SYF2, also known as p29 or Ntc31, has a long-known dual function in both spliceosome and cell cycle, from yeast to human (41). In budding yeast, SYF2 mutation affects splicing of genes encoding tubulin, leading to spindle checkpoint activation (42). In human, SYF2 has been involved in DNA damage-induced cell cycle checkpoint activation, and was proposed to control the replication checkpoint through an association with MCM3 and PCNA during S phase (43–45). However, little was known about the splicing events controlled by human SYF2. Our findings suggest that SYF2 promotes a slowdown of DNA replication and S-phase progression through AS regulation of ECT2-Ex5. Thus, SYF2 may promote the replication checkpoint and S-phase arrest (slowdown) through both splicing-dependent (this study) and independent mechanisms (43). Interestingly, within the spliceosome SYF2 is part of the yeast Prp19 complex and of the human Prp19-associated complex, two components of which also have a direct role in replication checkpoint activation or replication stress response (32,46–48). Thus, our findings may help understand the links between splicing regulation and cell-cycle checkpoint activation.

A more efficient replication checkpoint may favor replication-coupled repair and prevent cell death, as recently observed in cancer stem cells (49). It would be interesting to determine, whether the S-phase accumulation phenotype and the AS regulatory pathways (involving ZRANB2, SYF2 and ECT2-Ex5) that we identified, may be related to the cancer stem cell phenotype of Doxo-resistant cells (50).

At the gene expression level, with a cut-off of 2-fold, ZRANB2 and SYF2 depletion regulated 44 and 30 genes, respectively (Supplementary Figure S10A-B and Supplementary Table S8). There was no gene regulated at both AS and gene expression levels by ZRANB2, and only one in the case of SYF2, suggesting that the effects are not directly linked. None of the genes regulated at the gene level by either ZRANB2 or SYF2 encode splicing factors, suggesting direct effects on AS, and we provide evidence that ZRANB2 directly regulates AS of ECT2 and other targets. Conversely, for both ZRANB2 and SYF2, several genes regulated at the AS level are involved in transcription or RNA-stability regulation (Supplementary Tables S5 and S6), which might mediate their effects on gene expression levels. Consistently, SYF2 has not been involved in the regulation of either transcription or RNA stability. Thus, the 10 genes that were regulated in an opposite manner upon SYF2 depletion and resistance acquisition (Supplementary Figure S10B) are most likely indirect targets due to splicing effects on other genes. For ZRANB2, two studies have involved it in the regulation of transcription and RNA stability (51,52), therefore some gene-level regulation events observed upon ZRANB2 depletion may be direct. Of these, 19 were regulated in an opposite manner in DoxoR versus MCF-7 cells (Supplementary Figure S10A), and one of these 19 genes was regulated similarly by SYF2 depletion (Supplementary Figure S10C). Thus, while we provide evidence that AS of ECT2 plays a role in Doxo resistance (Figure 6A and Supplementary Figure S6C) and is directly regulated by ZRANB2 (Figure 5), we cannot exclude that gene-level regulation events (as well as additional AS events) might contribute to the effect of ZRANB2 on Doxo resistance. In addition, the lack of synergy between ZRANB2 and SYF2 depletion in regulating ECT2 Ex5 splicing and Doxo survival suggests that these two factors do not interact with each other, are not working in cooperation to regulate ECT2 Ex5 splicing, and that each factor might regulate additional targets contributing to Doxo resistance.

High mRNA levels of ZRANB2 and SYF2 did not associate with bad prognosis in our cohort of breast cancer patients treated with chemotherapy (data not shown). However, there is a frequent lack of correlation between RNA and protein levels for splicing factors (as exemplified by SYF2 in MCF-7 and MCF7-DoxoR cells). In fact, high protein levels of SYF2 were associated with bad prognosis in breast cancer (53), and we found that ZRANB2 mRNA levels were up-regulated 5.4 fold (*P* < 0.002) in a published dataset of non-responding versus responding breast tumors analyzed before chemotherapy using eprirubicin (54). Thus, the usefulness of ZRANB2 and SYF2 as predictive markers of chemotherapy resistance warrants further investigation. Interestingly, as ZRANB2 was found to be overexpressed in ovarian cancer and glioma (30,52), it would be interesting to assess, whether this overexpression is associated with resistance to therapy.

In the present study, we identify two convergent AS regulatory pathways (ZRANB2 and SYF2 splicing factors converging on ECT2 splicing) that control resistance to Doxo in breast cancer, specifically in ER+ERBB2- tumors (the main subtype of breast cancer). It is likely that other splice variants controlled by these splicing factors also play a role in Doxo resistance. Previous studies identified AS regulatory pathways (TRA2A → RSRC2, and PTBP1 → PKM) involved in resistance of triple-negative breast cancer to paclitaxel (a microtubule poison) and of pancreatic cancer to gemcitabine (a DNA synthesis inhibitor), respectively, as well as a splicing factor (hnRNPM) controlling an AS program and cell death response triggered by BEZ235 (a dual PI3K/mTOR inhibitor of potential clinical use) in Ewing sarcoma cells (15,16,55). Whether these AS pathways or programs intersect, and whether they are drug- or cancer type-specific, remain to be determined. Further identification of AS programs and regulatory pathways associated with and involved in anticancer drug resistance, should help understand the complex gene regulatory networks involved in resistance, taking into account multiple levels of gene expression regulation. This approach should also help identify novel biomarkers of resistance, and potential therapeutic targets.

## DATA AVAILABILITY

The datasets generated in this study have been deposited in the Gene Expression Omnibus repository (GSE126365, https://www.ncbi.nlm.nih.gov/geo/query/acc.cgi?acc=GSE126365) and in the UCSC genome browser (https://genome-euro.ucsc.edu/s/hpolveche/hg19_M%2DDUTERTRE_201901).

## Supplementary Material

gkz1213_Supplemental_Files

## Notes

Present address: Clara Benoit-Pilven, Institute for Molecular Medicine Finland (FIMM), University of Helsinki, Helsinki, Finland.
