# Supplementary material for: ZRANB2 and SYF2-mediated splicing programs converging on ECT2 are involved in breast cancer cell resistance to doxorubicin
Source: Nucleic Acids Res. 2020 Jan 16;48(5):2676–93. doi: 10.1093/nar/gkz1213 (PMC7049692; doi:10.1093/nar/gkz1213)
Supplement: gkz1213_Supplemental_Files [file gkz1213_supplemental_files.zip › SUP FIG S11-S12_FINAL FILE.pdf]

Figure S11: Sequence of ECT2.

Exon Intron

AGGTAA motif (ZRANB2-binding motif = two closely spaced AGGTAA motifs)

ZRANB2 binding sites (eCLIP dataset, G. Yeo, ENCODE)

PCR primer

AAAAATTAGGATAGATCTCAGATTTTTGGCTAGGATTTTTGTGGGAGGTGTGTTATGGTGGTGTTATAATTGAATATAGG
AAGAGAAATTGGTTGAGGGCAAAGGTGTGGAGAAGGGAGGTAGAATAATATTTATGACTACAGTTGAGTGTTTGTTGTAT
GTCTAGTAATGTTCTATGTGCTTTTGATACATTGTTATTTTTATTATATTCCTGTGAGTCAGGTATTAGTATGTTTATTT
TACAGAAAAAGAAACTGAGGCTCAGAGAAGGTCAGTGACTTTCTTTGGTTCATTAAAAAAATGGGTAATAATACTTTTTA
TAAATTCTAGGTAAAAAGAGCCTCTAATACTTACTTTTGCCCAATGACCATGTTTATGTATTTTTATTCAAATTTTATTT I2-E3
Ex3 TTTCAG**CTGATTTAGAAGAATACAAATCATGGCTGAAAATAGTGTATTAACATCCACTACTGGGAGGACTAGCTTGGCAG**
ACTCTTCCATTTTTGATTCTAAAGTTACTGAGATTTCCAAGGAAAACTTACTTATTGGATCTACTTCATATGTAGAAGGT
AAACCTGTTACCTGCTTTAAAACAATCAATTTCTATTTTAATTCTAAACTTATTAGTGACTTTCTTAAGATTTAATTTTA
ATACCAACTGTAATGCTAGAGCACAGGTATTAAGTAAATATTTGTTTACAGAATCCTTTTACACTATGTCATTTTTCTGA
TTATTATTTGGGTTATTTGTATGCTTTGCAGCTTTGAAAAGGGAGGCATCTTAATTTGGATATTCCTTTTTCTGTATGTG
AGGGCGGTCTAGCTACAGCATTATTAGCTAGAAGTTATACAGTCATGACTGGTTAGGTGAAAGCTACTTTTTCCTTTACC
ACTTTATAAATGGGTTTTTCCTAAAATTATATGCACTGTTGAGGAGGATGACTATGTAAGATAGCGCATTCTCTGGCTAG
GAGGAATGCTGATGTTTGTATACCCTGCTACAATATGGTTTCTCTTTCTCTAAAGTGATATATAGCCAGATATTTTTGTA I3-E4
ATAAGTTGGGAGTTGAGTCCTATTAAATTTAGAGAAAGTTATGATTCCCTCAAAGATGTAATACATACTAGATATAGCCA
Ex4 AAAGAGCGATAAGGACCTACTGATTGTGATGTTAATACATGATAAACATTGAAACATTTTACATTTTAACAG**AAGAGATG** I4-E5
CCTCAGATTGAAACAAGAGTGATATTGGTTCAAGAAGCTGGAAAACAAGAAGAACTTATAAAAGCCTTAAAGGTACGGAG
TTTTAGGTTTTAGTTTTTTTTGTAATGCAATTTTTCTTCCATCAGTGTGTAAATAGTTTCATAGCTTTCTTAACCTCATA I5_1
Ex5 (AS) CTTAAGTCTCTTTTCCACAG**ACTATTAAAATAATGGAAGTCCCTGTTATAAAGATAAAAGAAAGTTGTCCTGGAAAATCG**
GATGAAAAATTAATAAAAAGTGTTATTAATATGGTAGGTCAAAGTAACTCATTGCATGTGGCATGCTGCACTTCCCTTGA
TTGTATTATTCTACTTTCTGGTGATTGGAATTAGGCACAAGGTGTATTGTGTGCAATCTGAAGCTTAATGAATAGAAACT *I5_2*
GTTTTATACTTTTTAACTCCTTAGCTTCTTTTTTCTCATTCATTTTATATTTAGTCTAAATTCTTGCTGTATTTCCAAAA
TTATAAATCACGGTAATTTATAATCAGGGAATTTGCCTCAGCATACGAAATAATCTAATTCTGGAATCCAGCTGTTTTAG
TTTGCTAGGGCTGCCATAACAAAATACCACATAGTTAATTGCTTAAACAGTAGAAATTTCTTTTCTCACAGTTCTGGAGG
CTAGAAGTCCAAGATTAAGGTGTGTGTAGGTTTAGTTTCTCTTGAGGCCTCTCGCCTTGGCTTGCAGTGTCCTTTTGCTG I5.3C
TCCTCACATGGCCTTTCTTCTGTGTGTCCTTGCATCCCTGGTGTTTCCTCTTATTGGGACACCAGGTTGGATTAGGGCCC
ACCCCTATTATCTCATTTAACCTTAATTACCTCTTTAAAGTCCTTATCGCCAAATATAGTCACACTCTAAGGACTTCATT
CTTCATTCTAGGTTAGGGCTTCAACATGAATTTTGGGGGTACATAATTTAGCACATCACACTAGCCTTGCAAATAAAATT I5.3R
CAAATAATTTACTGACCCATTTTGAGTTAAAAAAAAATTTTTTTTTTAATTTAACACGAGTTACAAGAGTTATATAGGTA I5_4
CTTTAGTATCTTCATTATTTTTCGTGGATGTATAAAGTGTTTACTGTAAGTAACTTGCCTTTTTATATCCTACATTTGCC
ATGCTGCAACTGCTACATATTGCACATCTTTCTCATATATCTTACATATCTGCTAAAATGATTATGCTGATTTTCCTTTC I5_5
TGTAAGTATTAAGTGCTATAGTATTATTTTCCTATTTTTCTGAAGATAGTTAATTTGTACAATAAGAACTTTATGTATGG
TATCATTTTCTTGGTCAGTAGTGGATATCTTTCTAATGCATCTTGATATCAAAATGCATGTACTTAAAAACTTTAGCATT I5-E6
CTAATTTTATGGAACTTATGCATACTTGATGATTTATGACTAATAAGTAATAAGTCAGAATTATGCCTTTATAATACTGA
TCTTTAAAATTTAAGAACTTAATTTGAATGGAGATATGTATATGTAAAAATGTAAGTACGTAAAGAAATATGGTTGTTCA
TTAAGTGTAATGCTGATTATTGTGTGATAGAAACTTATTTTCTACAAATATAAACATGTTAAAGTTGATATTGAATTGAT
TGTTAAGCCTTATTTAGAGAGACAGATGGATTATTTGATATATAAATAATTTTTATTTCTGCTAACTATATGATGAAAAG
Ex6 **GACATTAAAGTGGGCTTTGTAAAGATGGAGTCAGTGGAAGAATTTGAAGGTTTGGATTCTCCGGAATTTGAAAATGTATT**
TGTAGTCACGGACTTTCAGGATTCTGTCTTTAATGACCTCTACAAGGCTGATTGTAGAGTTATTGGACCACCAGTTGTAT
TAAATTGTTCACAAAAAGGAGAGGTAAGCATATACATTTATTATGACTTTTCAAGTTAAAATTTTTATTAATGAATTTTA
ATTAGCAAAAAATTTATTTGGAAAAATCTCATTGATTGCTGAGAGCAAAATATTTAGAATAGGAATAATAGTGGCTATTC
TAATGTGGTCCTTTAATTCCTCCATTTTAAACTTAGTGGTGAAAAGCTAAGGCCTTTGAAAGTTAAATAATGATAACTGT
ACATATGCATATACACAGACAAATACATAGTCCTTTTTTTTTTTTTTTTTTTGAGACGGAGTCTTGCTCTGTGCCCAGGC
TGGAGTGCAGTGGCACGATCTCAGCTCACTGCAAGCTCCGCCTCCTGGGTTCACGCCAT

Figure S12: Sequence of MAST2.

Exon Intron

AGGTAA motif (ZRANB2-binding motif = two closely spaced AGGTAA motifs)

ZRANB2 binding sites (eCLIP dataset, G. Yeo, ENCODE)

PCR primer

Ex12

GGAATATTTCTTGCCCGAAAGTCATGTCCACCCTCCTCACAAGCAAACCTCACAGAGTTTTGTTTCTTTTCCCATCCACA
G**GTAACAGTCCTTTGGACAGCCCCCGGAATTTCTCTCCAAATGCACCTGCTCACTTTTCTTTTGTTCCTGCCCGTAG**GTA
AGTTGATAGGAAACCTCCTCTGGGACCAGCACATGTGGCACTTGCATGAGGGTTAATTGAATGTCAGATTCCTCCTTTAA
GTGTCACTACTCTTTCAAGTGTATTACCTGAAGCTCCCATCCATCAAAACTCTGTCTTTGCTTTCCTCTCCTAGGTTGTA
ATGATCGTGTTCTGGTTTTGCTTTATAGGTGCCCCATGTATTTGTGTTTGAATGTTTAAACAGGAGAGTAGATATTAAGT
AAAAGTAGATTTATTTGTATAATTCACTTTTTTTTTTAAGTCCCTGATGTCCTACGGGTATGTTCCAAAGCATTATACTC
TCAATTTAGAACTGTCCTACAGTGATGAATGAGAAAATAAGCTCTCCTCCAGGAGGACCCAAAGTTATACTCTTTGTCCT
CTCTAACTCAGCTAGTTTAAAGCAGCTGAAAGGGCTCTTTGTGCCTCTCTCCTTGACTTTGAGCTGGAGTTCGTTTGGGG
GATAAAGACACTACAGGGACTTCACTTTTCCTTCTATGAATTTAGAAAGATTTGGTGCATACAGAGCCATGCTTACAGTC
CTCCTCCCATGTTTCTTCCCTCTTGAGAGTGATAACAAACCTGTCTCTGGCCCATGGCAGCAACGCTGACAGCTCTGCCT
GTGGCTGGAATATCATCAGGCCTTTAGAGAAAGCTGAATGAGATTAATCCATCCAAATCACATACCTAGGCTCTATACAC
ACATGATTTCTTGTCAGAACTTTTCTAAGATTCTTTGCCAGAG*TTTATATTTATTTTATTTTATTTTTTAATTTTTAAAA*
*TT*GTTCTTGGTTAGAGTTAGTCCCTGGTATAAGACCTCCAGGAAAATTATGGTTCCAGATTTGTAACCTTTAATGGATGG
AGAATGTAATATAGCTGGAGACCAGGACAGTTTTAATACTTAGACTAC*TTTTTTTTTTTTTTTGAGACGGAGTCTCACTC*
TGTCACCCAGGCTGGAGTGCTGTGGCATGATCTCGGCTCACTGCAAACTCCGCCTCCCGGGTTCACGCCATTCTCCTGCC
TCAGCCTCCCGAGTAGCTGGGACTACAGGCGCCCGCCATCATTCCGAGCTAATTTCTTTTTTTGTATTTTTGATAGAGAC
GGGGTTCCACCGTGTTAGCCAGGATGGTCTTGATCTCCTGACCTCGTGATCCACCCGCCTCGGCCTCCCAAAGTGCTGGG
*ATTACAGGCATGAGCCACCGTGCCCGGCC*TACTTAGACTACTTTTTAAAAAAAGACATATTAGGAAAAAGAGAGGCTCCT
GGAAATGGTGTTAACTCACTATTCCAATGATAACCCAAAATAAATCCATCCTCTTTCCAGGATTCTTTGCAGCTGAAAAG
AACATGAACAGGTTGGGATAAGTTTGATAGCTGACGTGGGTTTCCTCCTTCAACCCACTTACCTTATTCAAAACAAATTT
TATCCAGGTTTGTTCCACGGGCTCATCTCTCCTACCCTATGGAACTTCCAATTTGTTAAGCAAGGATCCAGATAAGATCA
CTTACAGTATAAGAATATGCTGGTTTGATGCATTTGTTTGTCCAACCCAGCTGGAAAGACTTATCCCAGACTTCTTGCCT
GGACTATATATAAACTGTACTCTCAGAGTAGGTACGAGCTGGGACACCAAGACCTGTGGCTAGATCCCCATAGGTGGCAT
TGAGTTATTTC*TCATTCATTTGATAAATATTTGCTGAGCACCACCAACTAGAGTCAGGCGCACTCGGCTGGGTGCTTAGT*
TATGTAGTGGTAACAAAATAGACATAGTCCCTACTTCCCTGAAACTCAGGATGTAGGAGAGTCTGTGTACTGGTTGTTTC
CTCTGTAAGGAATGCTGTTCCCTCAGATCTTCATTTAGCTAACTCATTGTTATGATTCATATCTCATCTTAAATGTCACC
TCCTCAGAGAGGCCTTCCCTAAACATTCAATCTAAAGGGGCCTTCTTGTCTTTCTCCATCAAATCATCCAATTTTATTTT
*CAAACTAGTACTTTTCAATTCCTGATATTATCTTATTTGCATATTTTCTGTCTC*AATTCAACCACAGTCAAGTCACCTTT
AATAGAGATACCTTCATTTGTTGAAAGCTGTGTCCTGGCGGGAAGACAAATGAG*TAGTGGGAGAGAAAGACAAACAAATA*
TATGTTTATAAATTATGACAAGCACCATAGAGGGAAAGAGCATGATGCTGCATGAAAGAACAGGAAGAGGGCCGGGCACA
GTGGCTCACGCCTGTAATCCCAGCACTTTGGGAGGCCGAAGCAGGCATATCACTTGAAGTCGGGAGATTGAGACCAGCCT
GGCCAACATGGTGAAACACCATCTCTACTAAAAATACAAAAATTAGCCGGGCATGGTGGTGCAGGTCTGTAATCCTAGCT
GCTCGGGAGGCTGAGGCAGGAGAATCACTTGAATCCGGGAGGCAGAGGTTGCAGTGAGCCGAGATCGTGCCACTGCACTC
CAGCCTGGGCGACAGAGCAAGACTCCATCTGAAACAAAACAAAACAAAAAAAGAACAGGAAGAGACTAAAGCCAGAGAAC
CTCCACTGGGTGGCTATGTAAAGCCTCTCTGAAGGATGAGAAGCAGCTGCCATGTTAGGTGCGAGGACCCAGGTGTGTGT
*AGACCATTCCAGGCAGAGGGAACAGCCTGT*TCCATTGATACCATGGGTCTGTTTCATTGCCTGGGTTTAGGAGTAGGACC
AACTAGGAGGCTTCAGGGCCTGGTTTTTTACCACTGAGACCCAAACAGGTCTATGCTAAGTGGCATGACATTTGAGTCAT
GTGCATGGAACATGGATCTCTGGGCTGTCCTCTTATACAACATAGTAAGAAAAGCTGCTGGTTATTGGTGGGATGGGGAG
AAGTGTTTCAACCCTGCCAAACAGAGTATCAGGAATCGTTTGTTTCTGTGTTACTGGCTGGCGCTTTGGAGGATCACATT
GGTTTGCAATGTCAAACAAAAGACAGATTGAATTGAACACTAAGGATAGACCCATTGGTCCTACAATTTCACTTCAAACT
ATCACACATTCCTAAACAGAAGGAATGTGCCAAGCTAGACCAAATACCAAGATTCAAAAAGATCACTCTCTCTGGTTCCT

Ex13 (AS)

TTGGTTCTCACTATAATGTGTATTTTTTTACTACAG**CCATAGCCACAGAGCTGACAG**GTAATAGCGGGTATAATGGGGAG
AGGGGCTGGGAAACTGCTATGCATGGCCAGACAATATGGTTTGTGATGTTGTGGTTGGTGAAAACTGGTTGTGATGATTC
TTCTCTGACTGTGGGTATGAACATTGGGCCTTTCATGTCTTGCAAAGAGGCCCAAAGGGATCTCCCTCTACCTGCTGGAA
CCAAGGCTCAGAGCTCATATGCTTGGAGGCAGGAATTGCTGAATAGTTCCAAACTTCTGAGCTTAGGGACAAGTCACCCT
TATTCCCACAGCTTTGCACAGTCAGATGACTGCAGTGACCAGATAGAGGAAATAGAGGCATCATGTGGTGGCATTTCTTC
CTGACTCTGGTGGCCCCTGCTGTTCATTATACAGAGAAATTGCTGCCATGCAGCTAGTTTTCATTTCTATCTAAGGGGAG
GAAAACTCACATGTACAAAAGTGTATTTGACAAGGGTTTGGGAAATCCATCCCTGAGTTATACTTGTCTCATCTTTGCTG
AGCTATTTCCTGGAACCGACTTGTTTTATTGTGTGGTGCTCCCAGGACCCAGCAAAGAGGTCAAAGAGAACATCATGTCT
GAGCACCTTCCTGGACATGTCTGAACCTCAGAGACTAGGTTCCAGCAGAGTTATAGCCTATATGTGCTGCTGCCAGGGTG
GCATGAAGTTTGAGTCATGTGCATGTGGCATGGAACCTGCGGCTCACCCCTTACAGAACAGAGTAGAAAAGCTGCTAGGT
ATTGGTGAGGGTGGGGAGGAGTGCTTCGAAGGGAGAAGCCCCAGCAAGATTTATTCCTTTTTGCTTCTTCTTCTCCCTGT
CCCTGCCATAACCATGAAGCCTTGAACAAACCACCCAAATCTCAGGATCTTAGTGTTTTCTCTGTAAATTGTAATATGAA
CTTATAAAGATCCTCCATTGCTGATAGTCTCAGGTTCTGTGAGTAACAGCAAAAAAACTTTGTATCTAACTTCAACCAGA
GCAGGCTGTACCCTTAAGCTCTACAAAAAAGAATACACCACATCCCACAGTCCAGGCAAAGAATCACACGTTCATATTAT

AAAAGTTTCAAACTAGATCCCTGCTTGATCTTTCCCCTCTACAGTAAAGCAAATAACTGTGGCGGGGTGGGGGGGCATGT
TTTGTTTGTTTGTTTTGATGAGAGCTGTGGGGAGCATCCTTTTGAGAGCAGCAGGAAGCCTCCCCCAAAGTCTCATATAC
AGCTAAGGGTATGTCACCTCCCTTCTCTTTCATAAAGTTACTGTCACTCTTCGACAGACATTCCCAAATATTGGTTTTAT
GAGTCCTAACGGCTCCTAGGCAGCCTATAACTGTTTCCCATTGATTAGCTCAGTTCAGACCCAGATGGTGGCCCACTTCT
TCCTGTTGTCAGCCAGTGGCCTGGGGAAGACCAAAGAGCCCTTAACACGGAGCCTTCACAAATGGAAATAGCTGCATTTC
AGTGGCAGTTCTATCTTGTAATGTTGCCTATAGCTGGAATCCTCTGTCATTGTTTCTATGAAAGTAAGTTGAGAGTATGA
AAATATATATTCCAAAACCACAGTATTATATGACAGATGTTCTTCTGTTATACAAAGTTATGGACTATTTTATAGTCAGA
GATGTCTGTCTTTGCAGGGAAAACAACAATAAGCCCTCTGTAGGGGAGAGATATAAAAGCATGTACTGGACTGAGCAGTA
ATGTCTTAAGGAGGAGCCCTACAGTGAATCAACTGCCCCCAAAATGCTGATGAACAGACAGGCAGGTTGTGGGTCAGGGT
Ex14 GTAGTCCTGCAGAAACTGCCTAATACTTTTTCTTGCATACAG**GACTGATGGGCGGCGCTGGTCTTTGGCCTCTTTGCCCT**
CTTCAGGATATGGAACTAACACTCCTAGCTCCACTGTCTCAGTAAGTAGCCAAATCTGTGGCCATACTTCCTTCACCCTA
AGGAAGTACTTCCCTTTGGGTTATCTAGTGTCACAAAATGGTTCTCAACTTTTTCTCCAAAGTCAAACTCAGAGAGGTTG
CTGAGAGATGATGTTGGGTGCAAC
