# Supplementary material for: ZRANB2 and SYF2-mediated splicing programs converging on ECT2 are involved in breast cancer cell resistance to doxorubicin
Source: Nucleic Acids Res. 2020 Jan 16;48(5):2676–93. doi: 10.1093/nar/gkz1213 (PMC7049692; doi:10.1093/nar/gkz1213)
Supplement: gkz1213_Supplemental_Files [file gkz1213_supplemental_files.zip › Suppl Fig_S1-S10_FINAL FILE.pdf]

Figure S1

A.

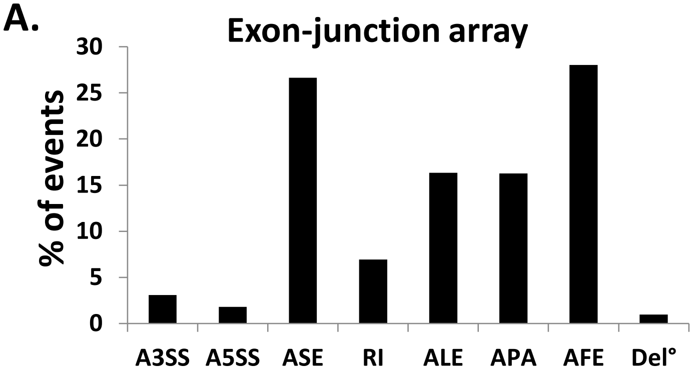

B.

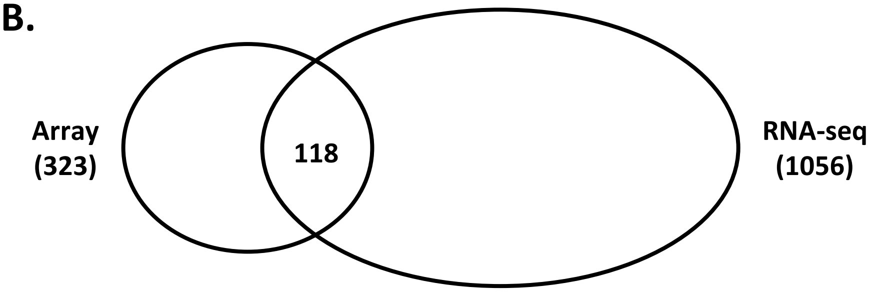

C.

Function annotation	p-value	# Genes
cytoskeleton	5,03E-07	80
cellular response to stress	1,51E-03	34
response to DNA damage stimulus	1,84E-03	25
DNA repair	3,48E-03	20
DNA metabolic process	3,67E-03	30
Cell Cycle, Mitotic	1,35E-02	19

D.

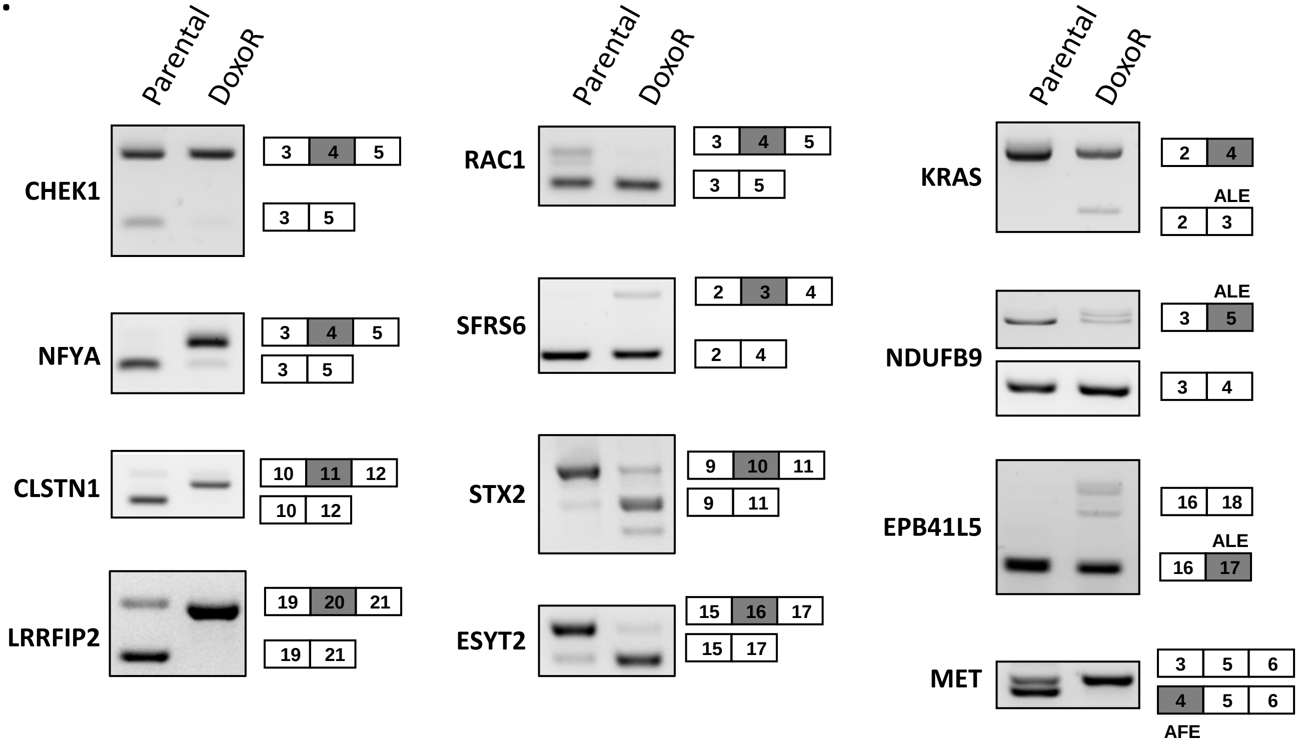

Figure S2

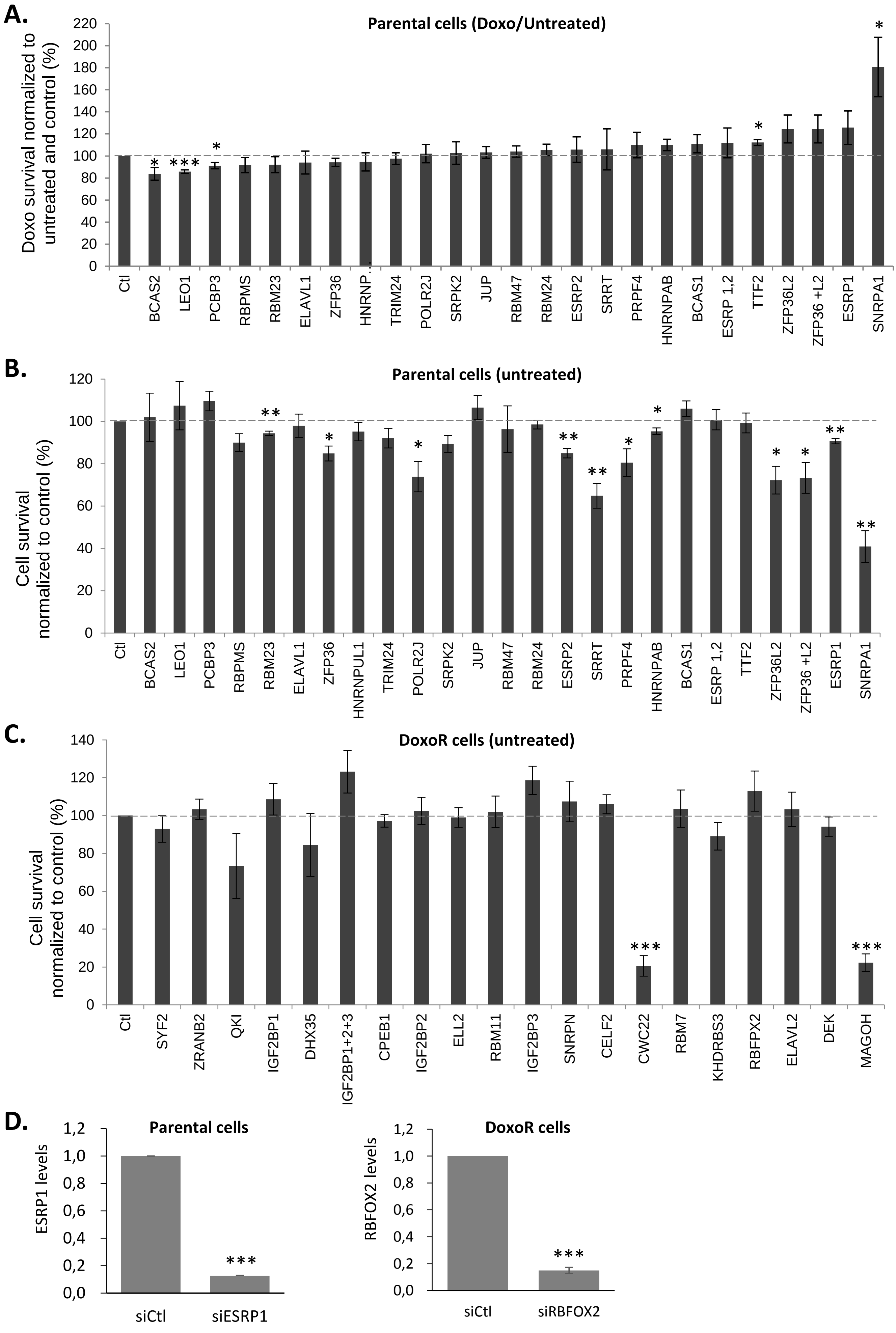

A.
Parental cells (Doxo/Untreated)
Doxo survival normalized to untreated and control (%)
0 20 40 60 80 100 120 140 160 180 200 220
Ctl BCAS2 LEO1 PCBP3 RBPMS RBM23 ELAVL1 ZFP36 HNRNP… TRIM24 POLR2J SRPK2 JUP RBM47 RBM24 ESRP2 SRRT PRPF4 HNRNPAB BCAS1 ESRP 1,2 TTF2 ZFP36L2 ZFP36 +L2 ESRP1 SNRPA1
B.
Parental cells (untreated)
Cell survival normalized to control (%)
0 20 40 60 80 100 120
Ctl BCAS2 LEO1 PCBP3 RBPMS RBM23 ELAVL1 ZFP36 HNRNPUL1 TRIM24 POLR2J SRPK2 JUP RBM47 RBM24 ESRP2 SRRT PRPF4 HNRNPAB BCAS1 ESRP 1,2 TTF2 ZFP36L2 ZFP36 +L2 ESRP1 SNRPA1
C.
DoxoR cells (untreated)
Cell survival normalized to control (%)
0 20 40 60 80 100 120 140
Ctl SYF2 ZRANB2 QKI IGF2BP1 DHX35 IGF2BP1+2+3 CPEB1 IGF2BP2 ELL2 RBM11 IGF2BP3 SNRPN CELF2 CWC22 RBM7 KHDRBS3 RBFPX2 ELAVL2 DEK MAGOH
D.
Parental cells
ESRP1 levels
0,0 0,2 0,4 0,6 0,8 1,0 1,2
siCtl siESRP1
DoxoR cells
RBFOX2 levels
0,0 0,2 0,4 0,6 0,8 1,0 1,2
siCtl siRBFOX2

Figure S3

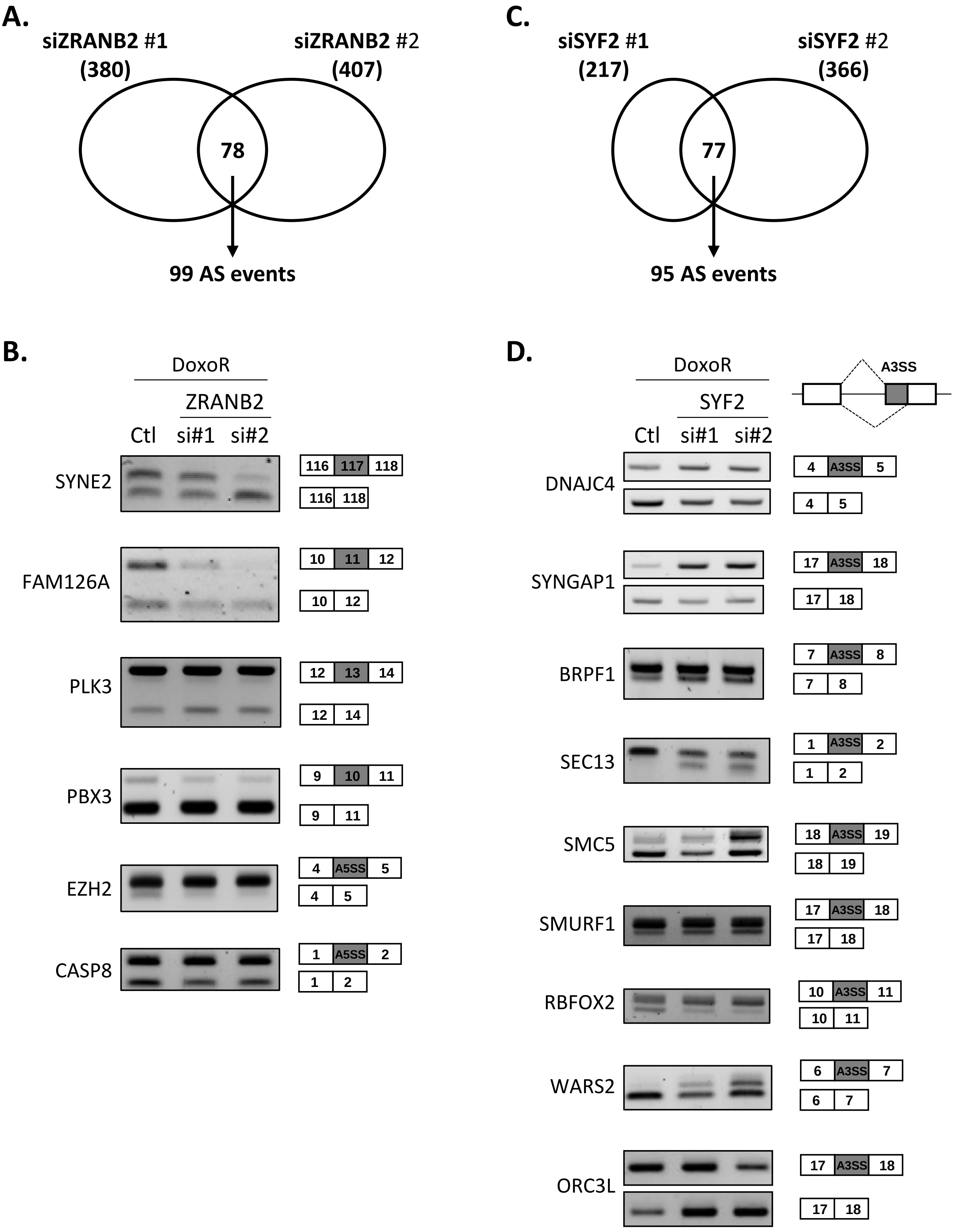
A.
siZRANB2 #1
(380)
siZRANB2 #2
(407)
78
99 AS events
B.
DoxoR
ZRANB2
Ctl
si#1
si#2
SYNE2
116 117 118
116 118
FAM126A
10 11 12
10 12
PLK3
12 13 14
12 14
PBX3
9 10 11
9 11
EZH2
4 A5SS 5
4 5
CASP8
1 A5SS 2
1 2
C.
siSYF2 #1
(217)
siSYF2 #2
(366)
77
95 AS events
D.
DoxoR
SYF2
Ctl
si#1
si#2
A3SS
DNAJC4
4 A3SS 5
4 5
SYNGAP1
17 A3SS 18
17 18
BRPF1
7 A3SS 8
7 8
SEC13
1 A3SS 2
1 2
SMC5
18 A3SS 19
18 19
SMURF1
17 A3SS 18
17 18
RBFOX2
10 A3SS 11
10 11
WARS2
6 A3SS 7
6 7
ORC3L
17 A3SS 18
17 18

Figure S4

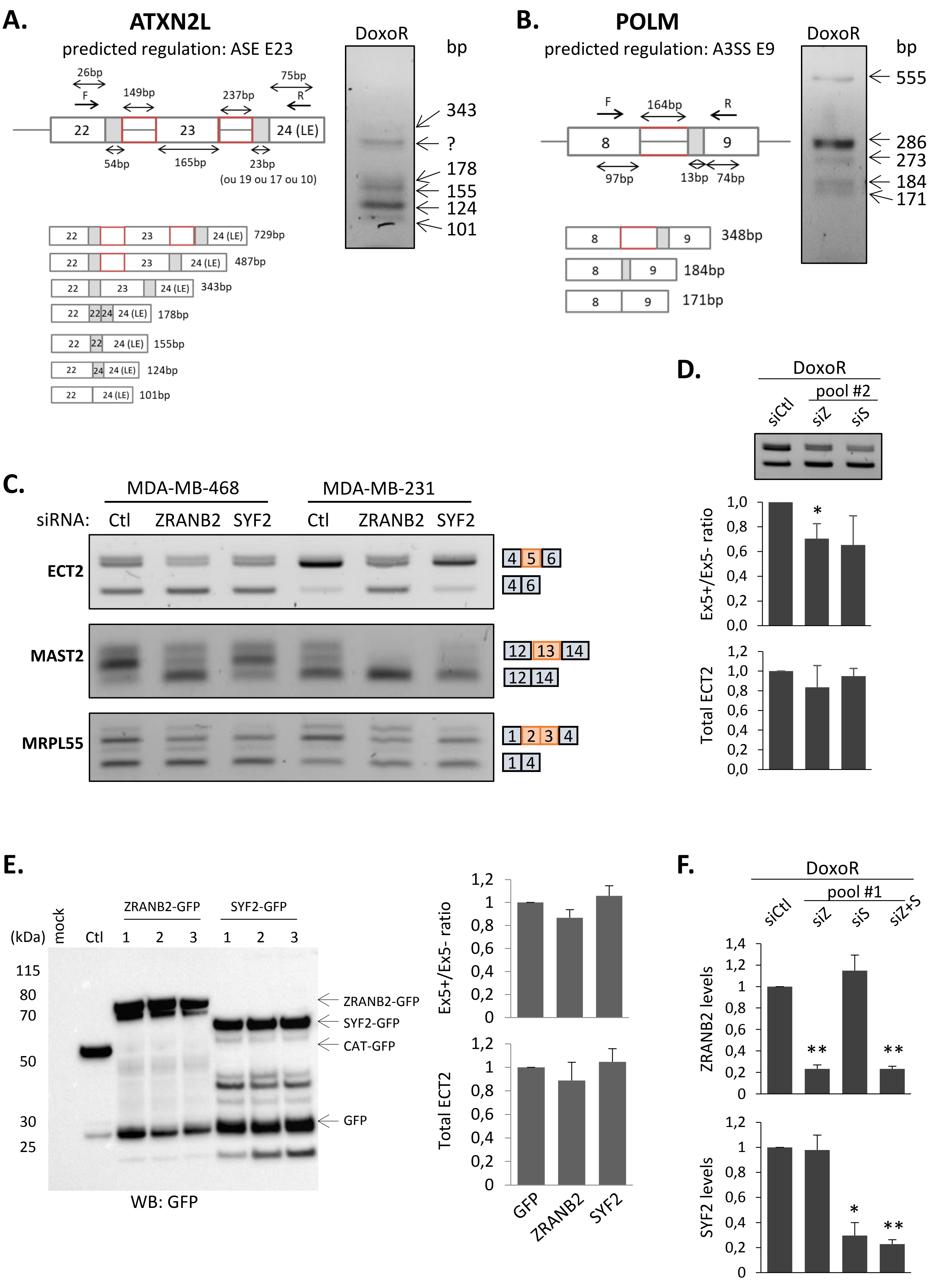
A.
ATXN2L
predicted regulation: ASE E23
26bp
F
149bp
237bp
75bp
R
22
23
24 (LE)
54bp
165bp
23bp
(ou 19 ou 17 ou 10)
729bp
487bp
343bp
178bp
155bp
124bp
101bp
DoxoR
bp
343
?
178
155
124
101
B.
POLM
predicted regulation: A3SS E9
F
164bp
R
8
9
97bp
13bp
74bp
348bp
184bp
171bp
DoxoR
bp
555
286
273
184
171
C.
MDA-MB-468
MDA-MB-231
siRNA: Ctl ZRANB2 SYF2 Ctl ZRANB2 SYF2
ECT2
MAST2
MRPL55
4 5 6
4 6
12 13 14
12 14
1 2 3 4
1 4
D.
DoxoR
pool #2
siCtl
siZ
siS
Ex5+/Ex5- ratio
Total ECT2
E.
(kDa)
mock
Ctl
ZRANB2-GFP
SYF2-GFP
1 2 3 1 2 3
115
80
70
50
30
25
ZRANB2-GFP
SYF2-GFP
CAT-GFP
GFP
WB: GFP
Ex5+/Ex5- ratio
Total ECT2
GFP
ZRANB2
SYF2
F.
DoxoR
pool #1
siCtl
siZ
siS
siZ+S
ZRANB2 levels
SYF2 levels

Figure S5

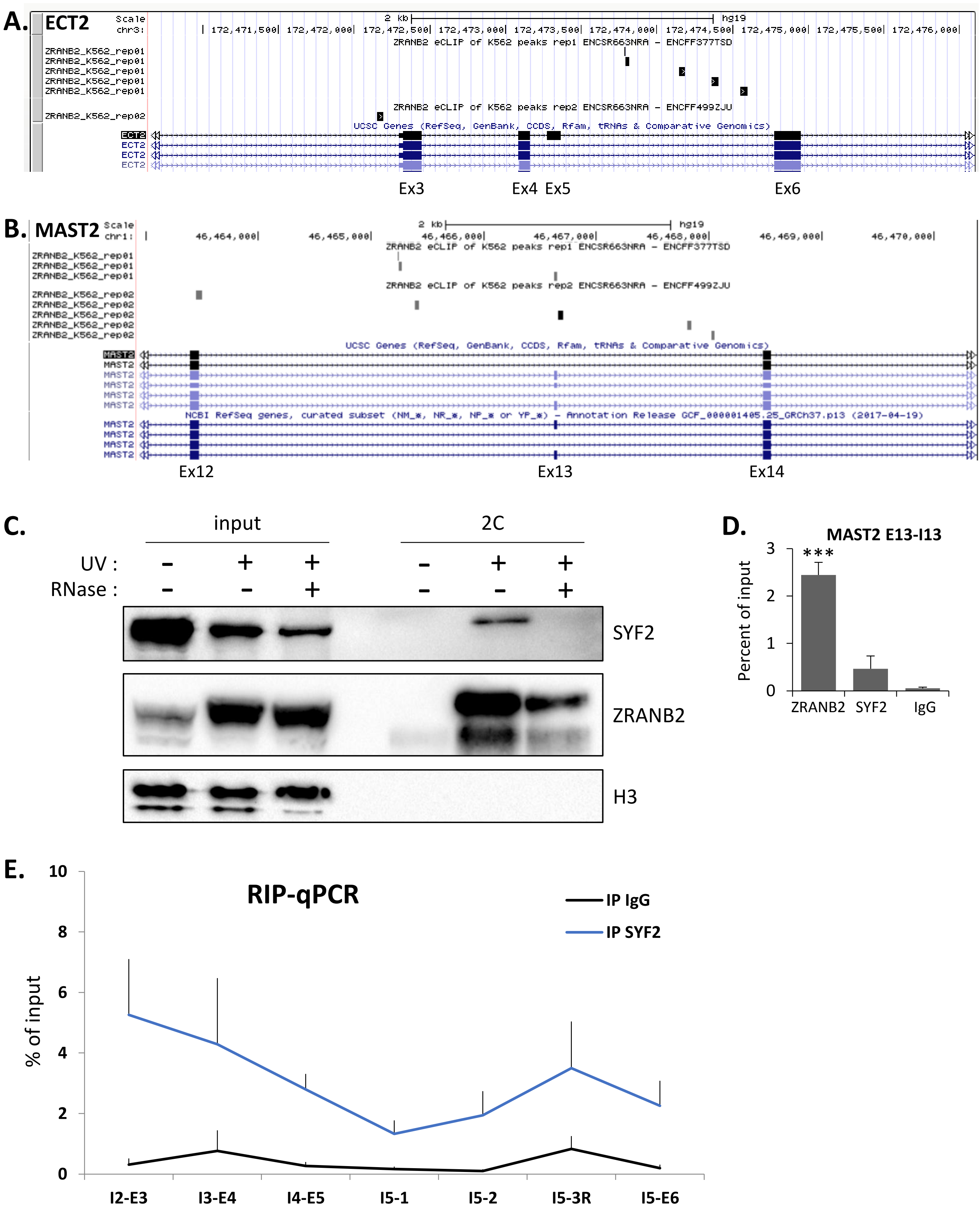

A.
ECT2
ZRANB2 eCLIP of K562 peaks rep1 ENCSR663NRA – ENCFF377TSD
ZRANB2 eCLIP of K562 peaks rep2 ENCSR663NRA – ENCFF499ZJU
UCSC Genes (RefSeq, GenBank, CCDS, Rfam, tRNAs & Comparative Genomics)
Ex3
Ex4
Ex5
Ex6
B.
MAST2
ZRANB2 eCLIP of K562 peaks rep1 ENCSR663NRA – ENCFF377TSD
ZRANB2 eCLIP of K562 peaks rep2 ENCSR663NRA – ENCFF499ZJU
UCSC Genes (RefSeq, GenBank, CCDS, Rfam, tRNAs & Comparative Genomics)
NCBI RefSeq genes, curated subset (NM_*, NR_*, NP_* or YP_*) – Annotation Release GCF_000001405.25_GRCh37.p13 (2017-04-19)
Ex12
Ex13
Ex14
C.
input
2C
UV :
RNase :
SYF2
ZRANB2
H3
D.
MAST2 E13-I13
Percent of input
***
ZRANB2
SYF2
IgG
E.
RIP-qPCR
IP IgG
IP SYF2
% of input
I2-E3
I3-E4
I4-E5
I5-1
I5-2
I5-3R
I5-E6

Figure S6

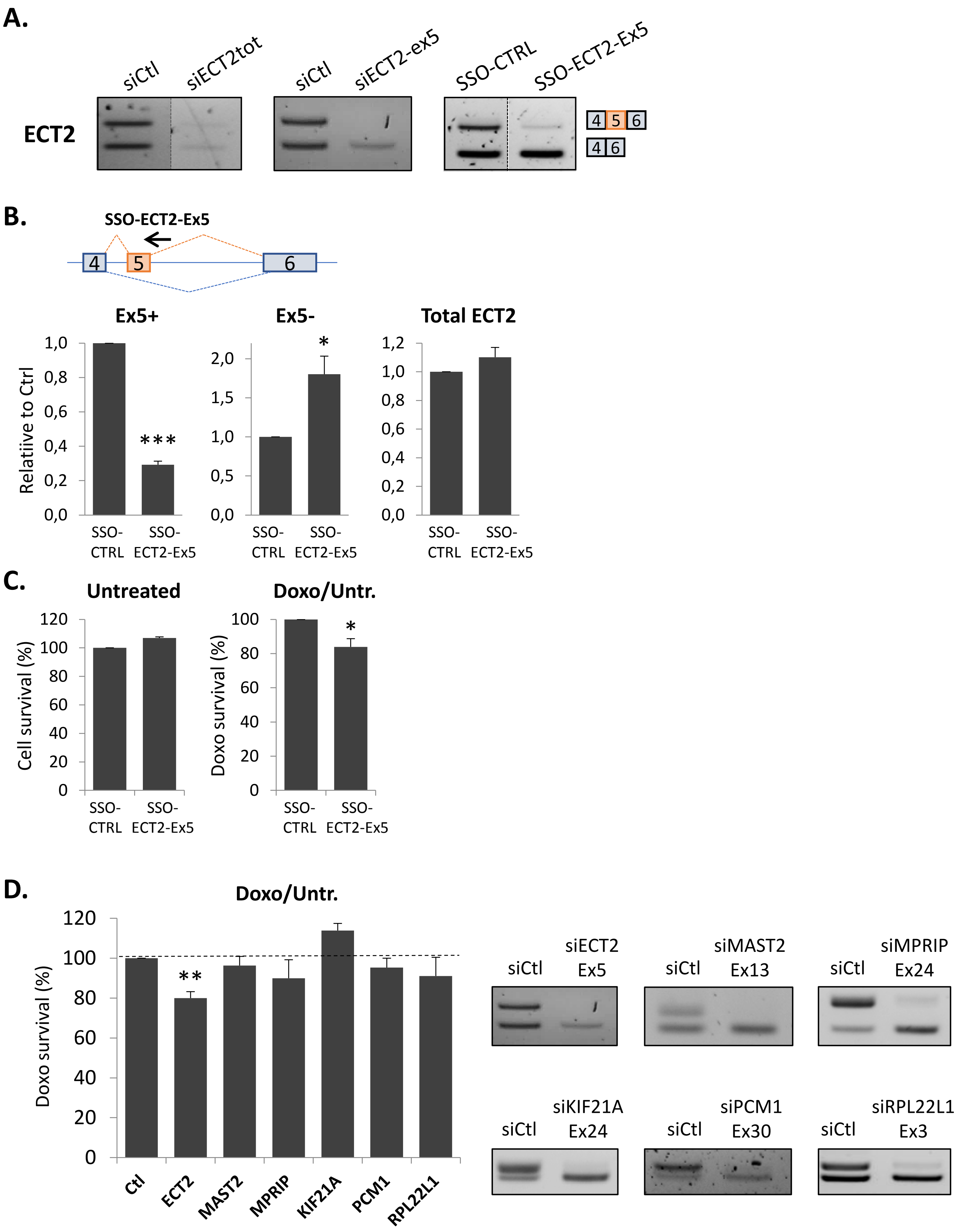

A.
siCtl
siECT2tot
siCtl
siECT2-ex5
SSO-CTRL
SSO-ECT2-Ex5
ECT2
4 5 6
4 6
B.
SSO-ECT2-Ex5
4
5
6
Ex5+
Ex5-
Total ECT2
Relatiive to Ctrl
1,0
0,8
0,6
0,4
0,2
0,0
***
2,0
1,5
1,0
0,5
0,0
*
1,2
1,0
0,8
0,6
0,4
0,2
0,0
SSO-CTRL
SSO-ECT2-Ex5
C.
Untreated
Doxo/Untr.
Cell survival (%)
120
100
80
60
40
20
0
Doxo survival (%)
100
80
60
40
20
0
*
SSO-CTRL
SSO-ECT2-Ex5
D.
Doxo/Untr.
Doxo survival (%)
120
100
80
60
40
20
0
**
Ctl
ECT2
MAST2
MPRIP
KIF21A
PCM1
RPL22L1
siCtl
siECT2 Ex5
siCtl
siMAST2 Ex13
siCtl
siMPRIP Ex24
siCtl
siKIF21A Ex24
siCtl
siPCM1 Ex30
siCtl
siRPL22L1 Ex3

Figure S7

A.

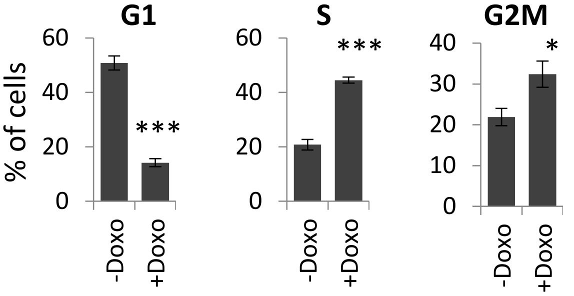

B.

siCtl

siZRANB2

siSYF2

siECT2-Ex5

siECT2tot

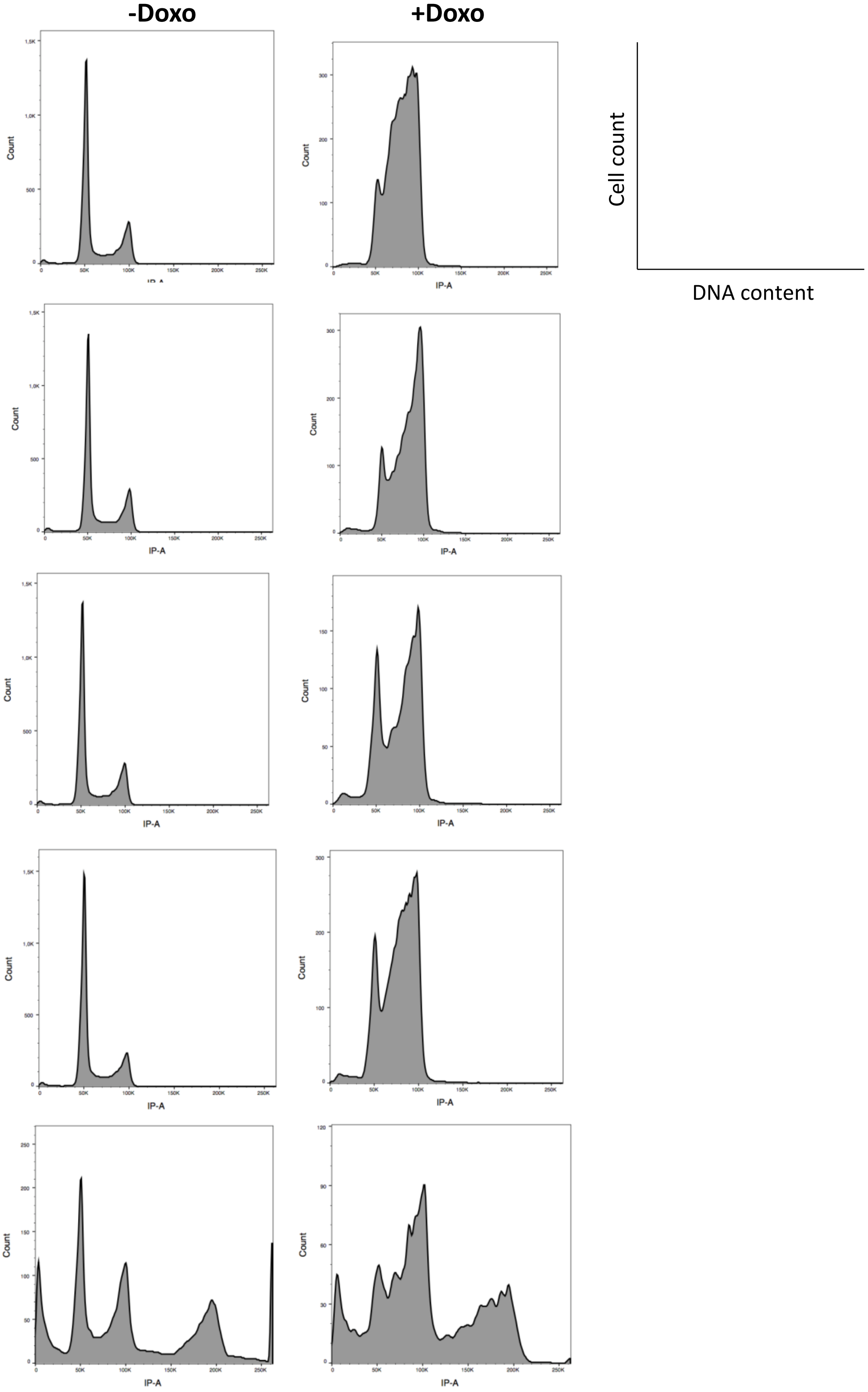

Figure S8

A. ECT2 Ex5+/Ex5- ratio in all subtypes of breast cancer

Patients with chemotherapy (n=237)

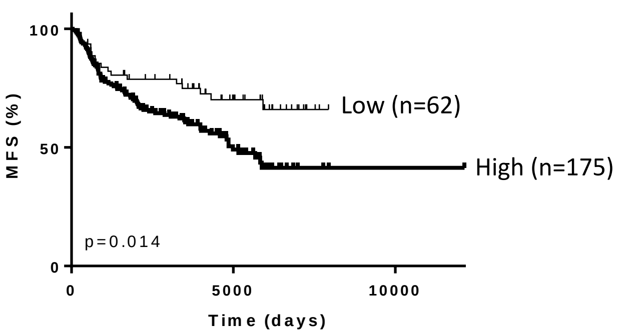

Patients without chemotherapy (n=289)

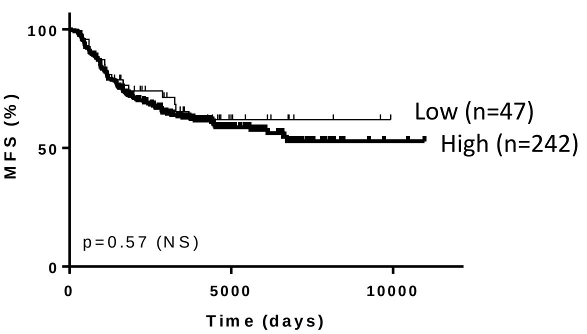

B. ECT2 Ex5+/Ex5- ratio in triple-negative breast cancer

Patients with chemotherapy (n=60)

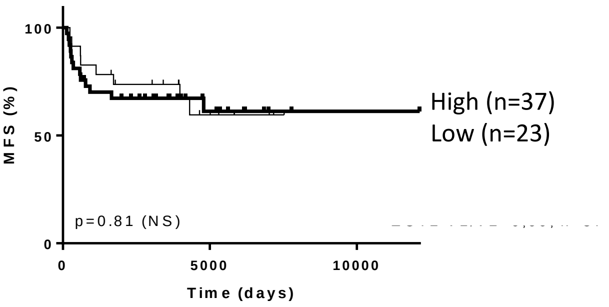

Patients without chemotherapy (n=41)

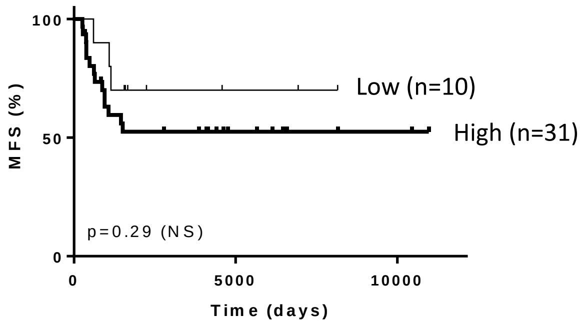

C. Total ECT2 mRNA levels in HR+ ERBB2- breast cancer

Patients with chemotherapy (n=120)

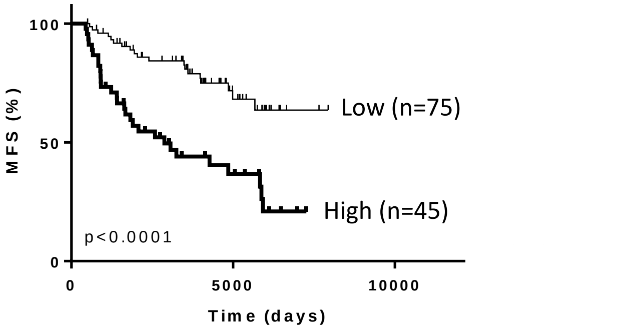

Patients without chemotherapy (n=174)

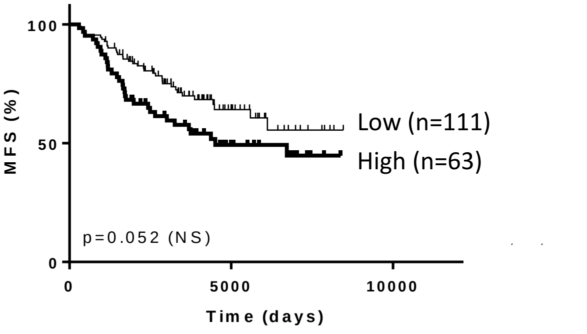

Figure S9

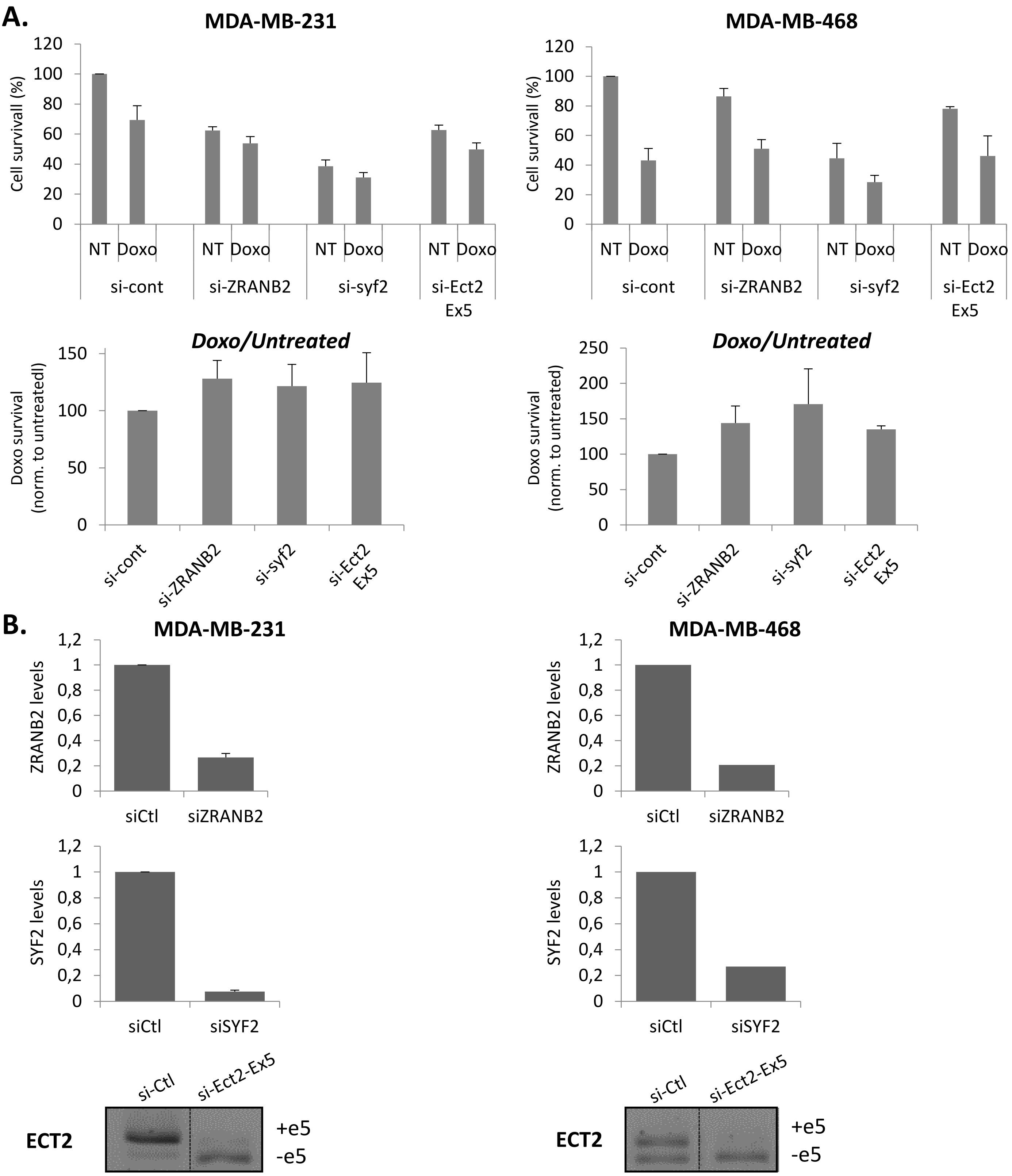

A.
MDA-MB-231
MDA-MB-468
Cell survivall (%)
120
100
80
60
40
20
0
NT
Doxo
si-cont
si-ZRANB2
si-syf2
si-Ect2 Ex5
Doxo/Untreated
Doxo survival (norm. to untreatedl)
Doxo survival (norm. to untreated)
250
200
150
100
50
0
B.
ZRANB2 levels
SYF2 levels
1,2
1
0,8
0,6
0,4
0,2
0
siCtl
siZRANB2
siSYF2
si-Ctl
si-Ect2-Ex5
ECT2
+e5
-e5

Figure S10

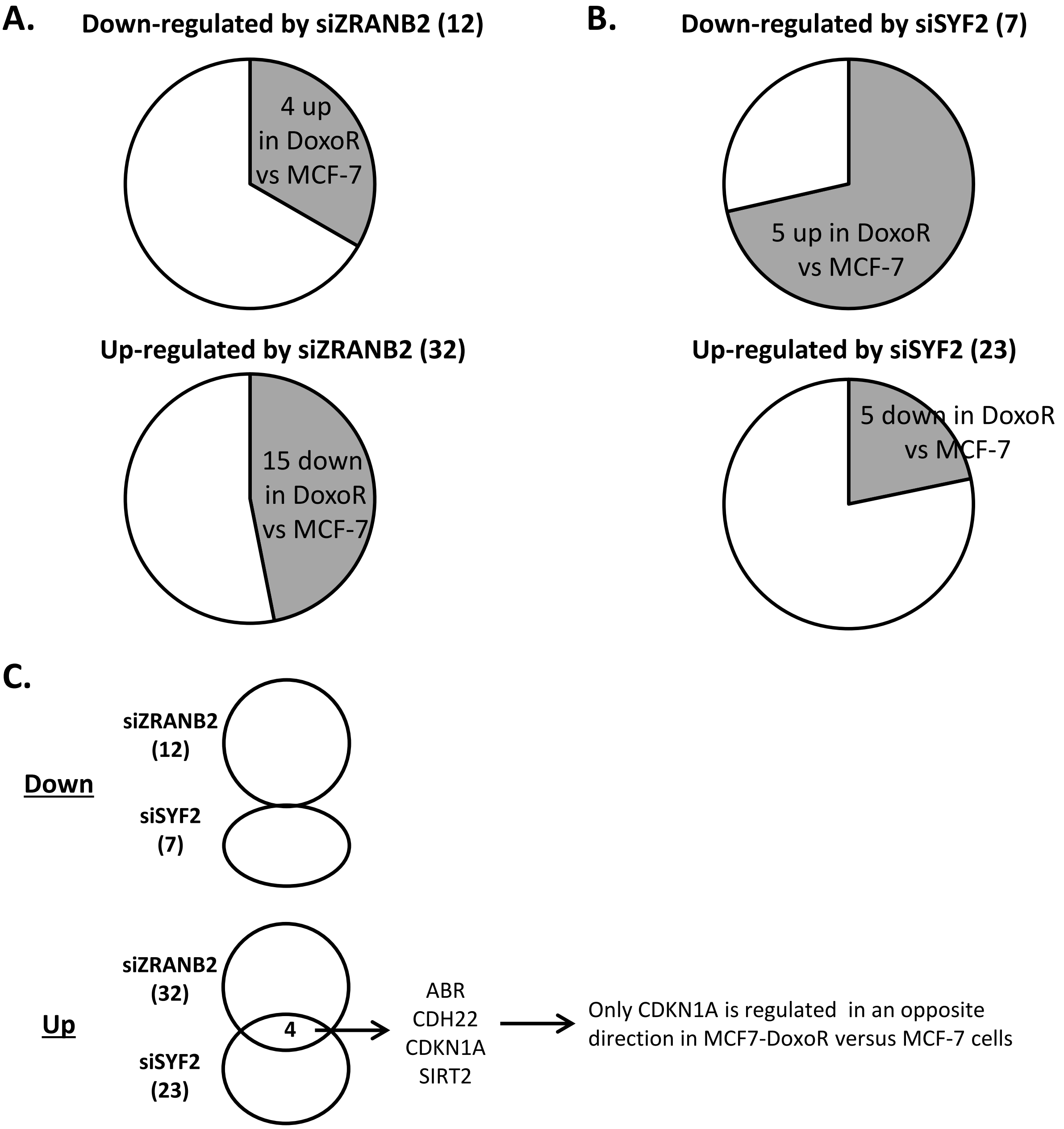
A.
Down-regulated by siZRANB2 (12)
4 up in DoxoR vs MCF-7
Up-regulated by siZRANB2 (32)
15 down in DoxoR vs MCF-7
B.
Down-regulated by siSYF2 (7)
5 up in DoxoR vs MCF-7
Up-regulated by siSYF2 (23)
5 down in DoxoR vs MCF-7
C.
Down
siZRANB2 (12)
siSYF2 (7)
Up
siZRANB2 (32)
siSYF2 (23)
4
ABR
CDH22
CDKN1A
SIRT2
Only CDKN1A is regulated in an opposite direction in MCF7-DoxoR versus MCF-7 cells
