# Supplementary material for: ZRANB2 and SYF2-mediated splicing programs converging on ECT2 are involved in breast cancer cell resistance to doxorubicin
Source: Nucleic Acids Res. 2020 Jan 16;48(5):2676–93. doi: 10.1093/nar/gkz1213 (PMC7049692; doi:10.1093/nar/gkz1213)
Supplement: gkz1213_Supplemental_Files [file gkz1213_supplemental_files.zip › SUPPLEMENTARY LEGENDS_FINAL FILE.pdf]

SUPPLEMENTARY FIGURE LEGENDS

Figure S1: **A**, Types of alternative exons regulated in MCF7-DoxoR *versus* parental MCF-7 cells in exon-junction array data. A3SS, alternative 3' splice sites. A3SS, alternative 3' splice sites. A5SS, alternative 5' splice sites. ASE, single-exon skipping (cassette exon). RI, retained intron. ALE, alternative last exon. APA, alternative polyadenylation. AFE, alternative first exon. MXE, mutually exclusive exons. **B**, Comparison of regulated cassette (ASE) exons found by RNA-seq and exon-junction array analyses. **C**, Enriched functions (Ingenuity Pathway Analysis) in genes with exonic regulation events in exon-junction array data. **D**, RT-PCR validations of exonic regulation events.

Figure S2: **A,** RNAi screen in MCF-7 cells. Following transfection and two-day recovery, cells were grown for three days with or without Doxo, and cell survival was assessed using a WST1 assay. For each siRNA, WST1 signal in the presence of Doxo was divided by WST1 signal in the absence of Doxo, and was expressed as percent of the data obtained with an siRNA that targets no gene (Ctl). **B-C**, Effects of splicing factor depletion on MCF-7 (B) and MCF7-DoxoR (C) cell survival without Doxo, as measured by WST1 assay. **D**, Validation of siRNA depletion of RBFOX2 and ESRP1 by RT-qPCR quantitation of their mRNA levels.

Figure S3: Overlap of genes regulated at the AS level by two siRNAs targeting ZRANB2 (A) or SYF2 (C) in MCF7-DoxoR cells transfected for 48 hours. RT-PCR validation of AS events regulated by ZRANB2 (B) and SYF2 (D).

Figure S4 : **A-B,** RT-PCR analysis of AS events in the *ATXN2L* and *POLM* genes in MCF7-DoxoR cells. **C**, RT-PCR analysis of AS regulation in the *ECT2*, *MAST2* and *MRPL55* genes in MDA-MB-468 and MDA-MB-231 cells. Validation of depletion is shown in Suppl. Fig. S9B. **D**, RT-qPCR analysis of ECT2 isoforms and total mRNA levels in MCF7-DoxoR cells transfected with siRNA pools #2 targeting ZRANB2 and SYF2 (whose depletion was validated in Fig. 2B-C). **E**, Analysis of MCF-7 cells transfected with GFP-tagged ZRANB2 and SYF2. Left, Western blot analysis with an anti-GFP antibody. Right, RT-qPCR analysis of ECT2 transcripts in FACS-sorted GFP+ cells. CAT-GFP, GFP-tagged chloramphenicol acetyltransferase (from a control plasmid). **F**, RT-qPCR analysis of ZRANB2 and SYF2 mRNA levels in MCF7-DoxoR cells transfected with siRNAs targeting ZRANB2 and SYF2.

Figure S5: **A-B**, ZRANB2 CLIP-seq data for the *ECT2* and *MAST2* genes (eCLIP dataset in ENCODE; ref. 31). Of note, the ZRANB2 binding site near ECT2-Ex5 was not detected in this CLIP-seq dataset, which was generated in blood cancer cells. **C**, Complex-capture analysis of SYF2 and ZRANB2 association with cellular RNA. See main text for details. **D,** CLIP-qPCR analysis of ZRANB2 and SYF2 association with MAST2 pre-mRNA at the indicated position. IgG, control immunoglobulin. **E,** RIP-qPCR analysis of SYF2 association with ECT2 pre-mRNA at the indicated positions.

Figure S6 : **A**, RT-PCR analysis of ECT2 isoforms. **B-C**, RT-qPCR analysis of ECT2 transcripts (B) and WST1 analysis of cell survival (C) in MCF7-DoxoR cells transfected with a splice-swicthing oligonucleotide targeting ECT2-Ex5 or a control oligonucleotide. **D**, WST1 analysis of Doxo survival (normalized to untreated

cells, left panel) and RT-PCR analysis of AS events (right panel) in MCF7-DoxoR cells transfected with siRNAs targeting the indicated alternative exons. WST1 data are presented as in Fig. 3.

Figure S7 : Cell cycle analysis by FACS with propidium iodide in MCF7-DoxoR cells. **A**, Quantitation of cells in G1, S and G2M phases in the absence or presence of Doxo 150 µM for 48 hours. **B**, FACS profiles of cells transfected with the indicated siRNAs and grown in the absence or presence of Doxo.

Figure S8 : Prognostic analysis of ECT2 Ex5+/Ex5- isoform ratio (A-B) and total ECT2 mRNA levels (normalized to TBP mRNA levels; C) in breast tumors before treatment. **A,** Breast tumors of all types. **B**, Triple-negative breast tumors. **C**, HR+ ERBB2- breast tumors. Patients were treated either with (left) or without (right) chemotherapy. The number of patients (n) and the p values are indicated. NS, not significant.

Figure S9 : Depletion of ECT2-Ex5, ZRANB2 and SYF2 does not affect Doxo survival in two triple-negative breast cancer cell lines. **A**, WST1 analysis of cell survival in the absence (NT) and presence of Doxo. **B**, RT-qPCR analysis of ZRANB2 and SYF2 mRNA levels, and RT-PCR analysis of ECT2 isoforms, showing the efficiency of depletion by siRNAs.

Figure S10 : RNA-seq analysis of genes regulated by at least 2-fold (up or down, as indicated) at the gene expression level by both siRNAs targeting ZRANB2 (A) or SYF2 (B) in MCF7-DoxoR cells. In grey, fraction of genes that are regulated at least 2-fold in the opposite direction in MCF7-DoxoR *versus* MCF-7 cells. **C**, Overlap between genes similarly regulated by ZRANB2 and SYF2 depletion.

Figure S11: Sequence of ECT2.

Figure S12: Sequence of MAST2.

SUPPLEMENTARY TABLES

Supplementary Table S1 (.xls): Sequences of PCR primers, siRNAs and morpholinos.

Supplementary Table S2 (.xls): Characteristics of the 526 breast tumors.

Supplementary Table S3 (.xls): Characteristics of the 120 HR+ERBB2- breast tumors treated with chemotherapy.

Supplementary Table S4 (.xls): List of exons regulated in MCF7-DoxoR versus MCF-7 cells (RNA-seq).

Supplementary Table S5 (.xls): List of exons regulated by ZRANB2 depletion in MCF7-DoxoR (RNA-seq).

Supplementary Table S6 (.xls): List of exons regulated by SYF2 depletion in MCF7-DoxoR (RNA-seq).

Supplementary Table S7 (.xls): Multivariate COX analysis of MFS for ECT2 Ex5+ / Ex5- ratio in a series of 120 HR+ERBB2- breast tumors treated with chemotherapy.

Supplementary Table S8 (.xls): Lists of genes globally regulated by ZRANB2 and SYF2 depletion in MCF7-DoxoR cells.
